# Tunable Fullerene Affinity of Cages, Bowls and Rings Assembled by Pd^II^ Coordination Sphere Engineering

**DOI:** 10.1002/chem.201903317

**Published:** 2019-10-24

**Authors:** Bin Chen, Shinnosuke Horiuchi, Julian J. Holstein, Jacopo Tessarolo, Guido H. Clever

**Affiliations:** ^1^ Faculty of Chemistry and Chemical Biology TU Dortmund University Otto-Hahn-Strasse 6 44227 Dortmund Germany; ^2^ Division of Chemistry and Materials Science Graduate School of Engineering Nagasaki University, Bunkyo-machi Nagasaki 852-8521 Japan

**Keywords:** coordination cages, fullerenes, molecular recognition, self-assembly, supramolecular chemistry

## Abstract

For metal‐mediated host compounds, the development of strategies to reduce symmetry and introduce multiple functionalities in a non‐statistical way is a challenging task. We show that the introduction of steric stress around the coordination environment of square‐planar Pd^II^ cations and bis‐monodentate nitrogen donor ligands allows to control the size and shape of the assembled product, from [Pd_2_L_4_] cages over [Pd_2_L_3_] bowl‐shaped structures to [Pd_2_L_2_] rings. Therefore, banana‐shaped ligand backbones were equipped with pyridines, two different quinoline isomers and acridine, the latter three introducing steric congestion through hydrogen substituents on annelated benzene rings. Differing behavior of the four resulting hosts towards the binding of C_60_ and C_70_ fullerenes was studied and related to structural differences by NMR spectroscopy, mass spectrometry and single crystal X‐ray diffraction. The three cages based on pyridine, 6‐quinoline or 3‐quinoline donors were found to either bind C_60_, C_70_ or no fullerene at all.

## Graphical abstract

**The introduction of steric stress** around the coordination environment of square‐planar Pd^II^ cations allows to control the size and shape of the assembly product, from [Pd_2_L_4_] cages over [Pd_2_L_3_] bowl‐shaped structures to [Pd_2_L_2_] rings with distinctive fullerene binding abilities.

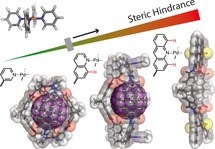

## Introduction

The metal‐mediated self‐assembly of supramolecular host systems with nano‐sized cavities has been extensively explored in the last decades. Numerous examples based on the combination of different donors and transition or main group metal cations have been reported, including pioneering work by Fenske, Fujita, Jin, Lehn, Nitschke, Puddephatt, Raymond, Saalfrank, Shionoya, Stang, Süss‐Fink, Ward and others.^1^ The combination of palladium(II) cations with pyridine‐based ligands turned out to be a very fruitful sub‐area with a recent upsurge in contributions by Chand, Crowley, Lusby, Lützen, Sallé, Severin, Yoshizawa, our group and other researchers.^2^ Most reported examples consist of one type of ligand, each, assembling with *cis*‐protected or “naked” Pd^II^ cations into rings, cages and spheres of rather high symmetry. With dimensions on the nanometer scale, the cavities enclosed by the supramolecular architectures find application as selective receptors and reaction environments.^3^ Chemical transformations under confinement were shown to be accelerated by proximity and local concentration effects, the (de)stabilization of specific ground and transition state geometries and the creation of a fine‐tuned electrostatic and pH milieu.^4^ Furthermore, various defined functionalities have been incorporated into the organic backbone structures of such assemblies, including photo‐switches, redox‐active sites, chiral groups and catalytic moieties.^5^

When comparing the majority of reported host systems to biological nano‐confinements such as enzyme pockets, the pronounced difference in symmetry and functional makeup of the cavities is striking. Therefore, quite some recent activity in the area of palladium‐mediated assembly is dedicated to the rational construction of less symmetric architectures, allowing the incorporation of multiple functions.^6^ Some approaches make use of specific interactions between ligand backbones or ligands and guests to control self‐sorting in mixed‐ligand systems.^7^ We have recently introduced a family of heteroleptic [Pd_2_L_2_L′_2_] cages in which the integrative self‐sorting of shape‐complementary ligands leads to clean product formation under thermodynamic control.^8^ Related examples were reported by Mukherjee and Chand.^9^ Further strategies base on the non‐statistical construction of heteroleptic coordination environments by engineering the direct electronic or steric environment of the metal coordination site.^10^ Fujita and Yoshizawa used pyridine/lutidine pairs around *cis*‐protected Pd^II^ to construct prismatic cages,^11^ and we recently expanded this concept to work on “naked” Pd^II^ centers, using a combination of inward and outward pointing picolines to give *cis*‐[Pd_2_L_2_L′_2_] cages.^12^ Crowley achieved similar structures with 2‐amino‐modified pyridine ligands.^13^

When considering other sterically more demanding donor functionalities, we recently started to substitute pyridines with quinolines, which carry an hydrogen atom on the annelated benzene ring that causes steric congestion around the metal binding site.^14^ We tested this method on bis‐monodentate banana‐shaped ligands with a dibenzo‐2.2.2‐bicyclo‐octane backbone and showed that the pyridine derivative **L^1^** leads to the formation of a typical [Pd_2_ **L^1^**_4_]^4+^ cage while 6‐quinolinyl derivative **L^2^** cleanly forms bowl [Pd_2_ **L^2^**_3_(MeCN)_2_]^4+^ when palladium precursor [Pd(MeCN)_4_](BF_4_)_2_ was mixed with **L^2^** in a 2:3 ratio in acetonitrile.^15^ As explanation for the latter finding, we indeed identified steric congestion around the coordination site, as observed in the single crystal X‐ray structure of the bowl‐shaped compound. While this situation disfavors (but not absolutely prevents) the binding of a fourth bis‐monodentate quinoline ligand, the square‐planar coordination sphere of the Pd^II^ cations is either completed by an acetonitrile molecule or a chloride ligand.

**Figure 1:**
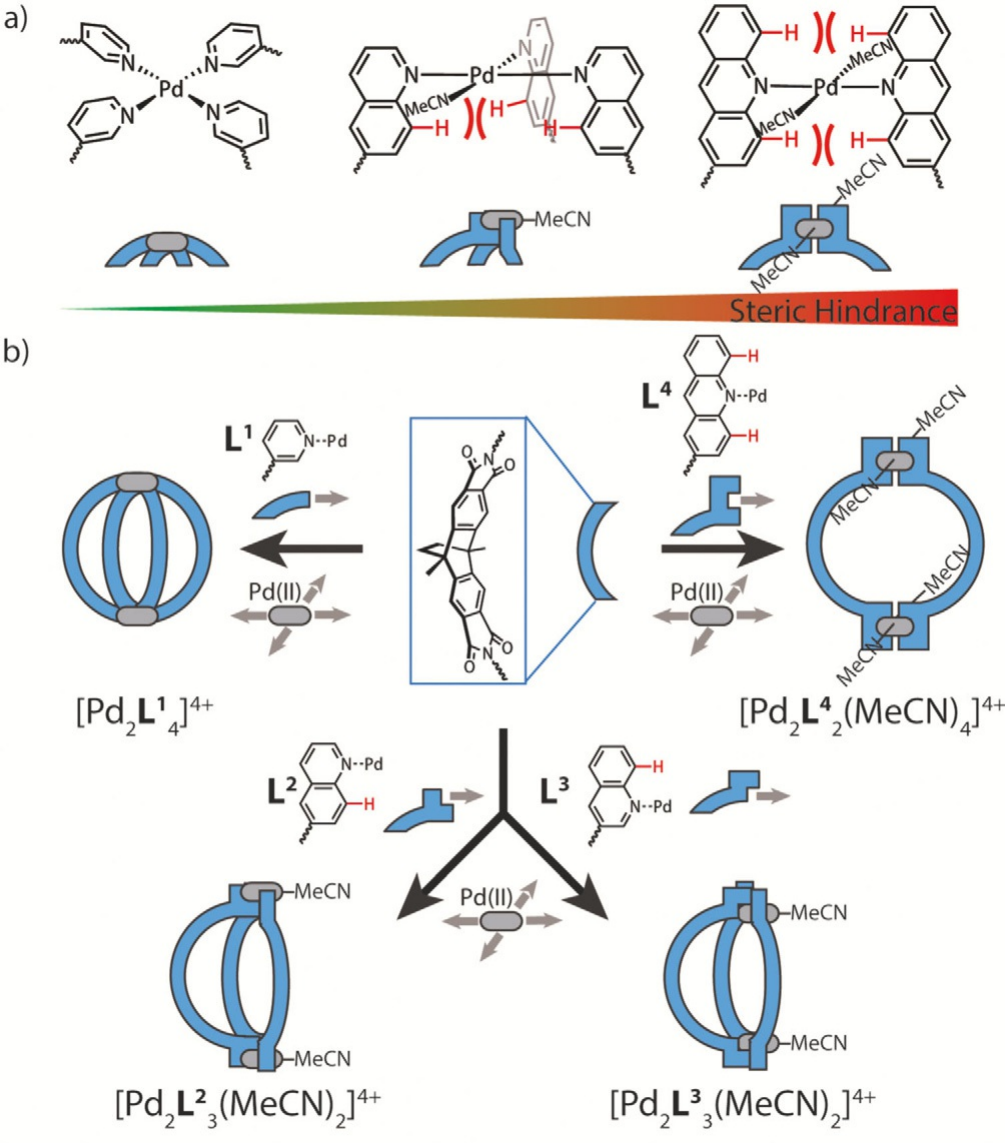
Self‐assembly of cages, bowls and rings under control of steric congestion in the Pd^II^ coordination sphere. (a) Increasing steric demand in the order pyridine, quinoline and acridine determines the number of nitrogen heterocycles in the coordination sphere of the dinuclear assemblies. (b) Modular functionalization of a curved backbone to give ligands **L^1^**‐**L^4^**, reacting with Pd^II^ into cage [Pd_2_ **L^1^**_4_]^4+^, isomeric bowls [Pd_2_ **L^2^**_3_(MeCN)_2_]^4+^ and [Pd_2_ **L^3^**_3_(MeCN)_2_]^4+^ and ring [Pd_2_ **L^4^**_2_(MeCN)_4_]^4+^.

Here, we expand the coordination site engineering principle onto new 3‐quinolinyl (**L^3^**) and 2‐acridinyl (**L^4^**) ligands with the former one being an isomer of **L^2^** that can also form a [Pd_2_ **L^3^**_4_]^4+^ cage as well as a bowl structure [Pd_2_ **L^3^**_3_(MeCN)_2_]^4+^, albeit with a different guest preference than the **L^1^**‐ and **L^2^**‐based systems. Ligand **L^4^**, on the other hand, brings in two sterically demanding C−H groups, on either side of the coordinating nitrogen atom, thus further enhancing congestion and leading to the exclusive formation of ring [Pd_2_ **L^4^**_2_(MeCN)_4_]^4+^ (Figure 1).

As we reported before, the backbone of the ligands was designed as a curved combination of two π‐surfaces to bestow the hosts with the ability to bind fullerenes and solubilize them in polar organic media.^15^ Furthermore, the bowl‐shaped compound was found to act as a supramolecular protecting group to allow the selective monofunctionalization of its bound fullerene guest. In a similar way, the herein presented cage, bowl and ring derivatives were in part found to bind fullerenes C_60_ and C_70_. Intriguingly, the tendency of the isomeric quinoline ligands to form bowls or cages as well as the fullerene affinities of all examined cage and bowl systems in comparison show more variety than initially expected and we herein suggest a number of structural reasons to explain these observations.

## Results and Discussion

### Bowl and cage assembly

**Figure 2:**
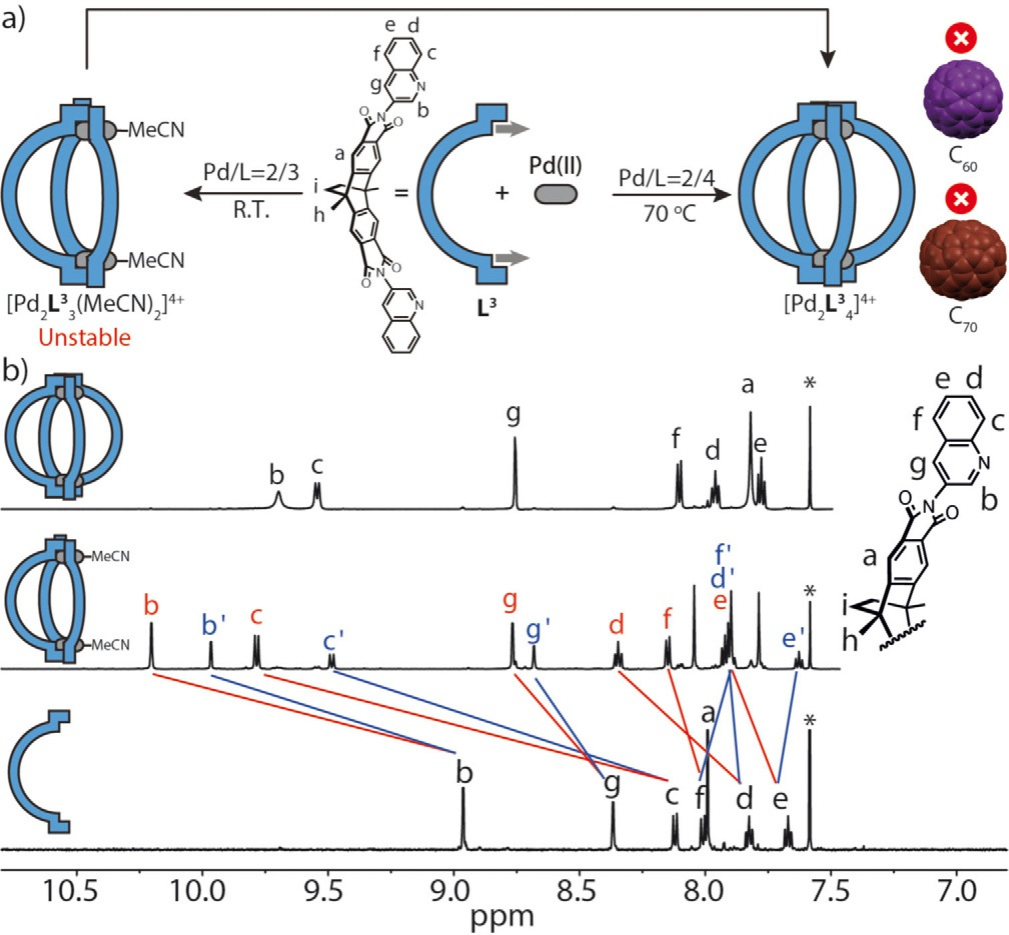
Self‐assembly and characterization of bowl‐shaped [Pd_2_ **L^3^**_3_(MeCN)_2_]^4+^ and cage [Pd_2_ **L^3^**_4_]^4+^. (a) **L^3^**, comprising sterically demanding hydrogen substituent H_c_, reacts with Pd^II^ to form bowl [Pd_2_ **L^3^**_3_(MeCN)_2_]^4+^ in 3:2 stoichiometry at room temperature, but forms cage [Pd_2_ **L^3^**_4_]^4+^ (4:2 stoichiometry) at 70 °C. In contrast to [Pd_2_ **L^1^**_4_]^4+^, cage [Pd_2_ **L^3^**_4_]^4+^ is not able to bind fullerenes. (b) ^1^H NMR spectra (600 MHz, 298 K, CD_3_CN) of ligand **L^3^**, bowl [Pd_2_ **L^3^**_3_(MeCN)_2_]^4+^ (0.64 mm) and cage [Pd_2_ **L^3^**_4_]^4+^(0.64 mm) from bottom to top. Signals assigned to edge/central ligands in the bowl are marked in red/blue.

The synthesis of backbone dianhydride has been described before.^16^ It can be readily modified with different nitrogen donors, here by reacting it with 3‐aminoquinoline/2‐aminoacridine to obtain ligands **L^3^** and **L^4^**, respectively. Compared to reported quinoline donor **L^2^**, the new quinoline ligand **L^3^** carries a protruding hydrogen substituent on the outer face of the metal‐coordinating nitrogen atom (with respect to the host's center). It was considered to exhibit a similar behavior in self‐assembly as **L^2^**, thus forming a bowl‐shaped structure in which the coordination of three quinolines to each Pd^II^ cation is supplemented by a solvent molecule as the fourth ligand. Indeed, bowl [Pd_2_ **L^3^**_3_(MeCN)_2_]^4+^ was quantitatively formed by stirring a 3:2 mixture of **L^3^** and [Pd(MeCN)_4_](BF_4_)_2_ in deuterated acetonitrile for 2 hours at room temperature, verified by NMR spectroscopy and mass spectrometry. Unlike the corresponding bowl formed by **L^2^**, however, compound [Pd_2_ **L^3^**_3_(MeCN)_2_]^4+^ exhibited instability even at room temperature, partially converting to cage [Pd_2_ **L^3^**_4_]^4+^ over the course of 2 d (Figure S12). Heating the freshly prepared bowl sample at 70 °C leads to a complete structural reorganization into cage [Pd_2_ **L^3^**_4_]^4+^ after 24 h (Figures S13 and S14). The ^1^H NMR spectrum of freshly prepared bowl [Pd_2_ **L^3^**_3_(MeCN)_2_]^4+^ revealed a downfield shift of most proton signals, attributed to metal complexation (Figure 2 b). The quinoline ^1^H signals were found to split into two sets with 2:1 integral ratio, in accordance with the reduced geometry. In addition, the bowl stoichiometry was further supported by the observation of prominent peaks in the ESI mass spectrum (Figure S11), consistent with the formula [Pd_2_ **L^3^**_3_(MeCN)_2_]^4+^ and [Pd_2_ **L^3^**_3_+BF_4_]^3+^, alongside three small signals of cage [Pd_2_ **L^3^**_4_+*n*BF_4_]^4−*n*+^ (*n*=0–2), most probably resulting from partial reorganization of the thermodynamically unstable bowl species.

Cage [Pd_2_ **L^3^**_4_]^4+^ could indeed be obtained quantitatively by heating a 2:1 mixture of **L^3^** and [Pd(MeCN)_4_](BF_4_)_2_ at 70 °C for 2 d, which is in pronounced contrast to what was observed for **L^2^**, where corresponding cage [Pd_2_ **L^2^**_4_]^4+^ only arose as a minor product under the same conditions. A comparison of DFT‐calculated energies of the bowl/cage equilibria for **L^2^** and **L^3^** supported this experimental observation by showing that bowl [Pd_2_ **L^2^**_3_(MeCN)_2_]^4+^ is the favored species for **L^2^**, while cage [Pd_2_ **L^3^**_4_]^4+^ is the thermodynamic minimum for **L^3^** (Figure S37 and S38). Cage [Pd_2_ **L^3^**_4_]^4+^ formed as a stable, single species in solution, as identified by NMR spectroscopy and ESI mass spectrometry (Figure 2 b and Figure S17). Suitable crystals for X‐ray analysis were obtained from the diffusion of methyl tert‐butyl ether into an acetonitrile solution of the cage, yielding a *C*_4_‐symmetric, helically twisted geometry (Figure 4 b). The latter fact can be attributed to the quinoline donors adjusting themselves around the Pd^II^ coordination center in a pronounced propeller shape that reduces the steric hindrance between the four squeezed hydrogen atoms of the annelated benzene rings. A mean distance of 2.82 Å was found between adjacent hydrogen atoms H_c_. Correspondingly, the Pd−Pd distance elongated to 16.19 Å along with a decrease of the VOIDOO‐calculated cavity volume (518 Å^3^), compared with the original pyridine cage [Pd_2_ **L^1^**_4_]^4+^ (572 Å^3^). Most interestingly, cage [Pd_2_ **L^3^**_4_]^4+^ has no ability to bind fullerenes, neither C_60_ nor C_70_ and no matter whether the guests are offered before or after cage formation. In comparison with fullerene‐binding cage [Pd_2_ **L^1^**_4_]^4+^, this is remarkable, since both cages share exactly the same inner chemical structure (and the same number of atoms between the coordinating nitrogen atoms within the ligands) and only differ by the absence/presence of the annelated benzene rings outside the guest‐binding cavity. It is further worth noting, that a 1:1:1 mixture of ligands **L^1^**, **L^3^** and Pd^II^ cations leads to a statistical cage mixture [Pd_2_(**L^1^**)_*n*_(**L^3^**)_4−*n*_] with *n*=0–4.

### Ring assembly

**Figure 3:**
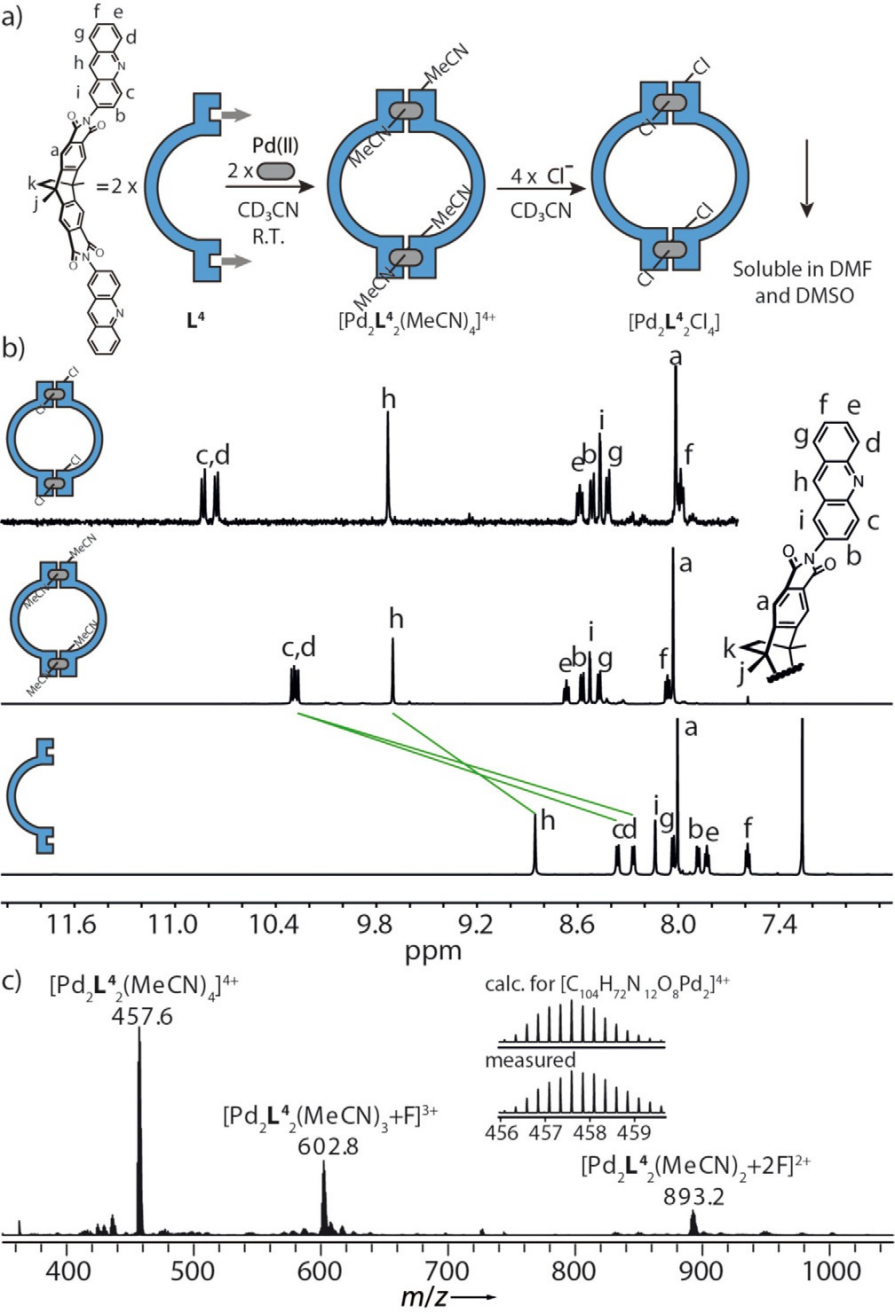
Self‐assembly and characterization of ring‐shaped [Pd_2_ **L^4^**_2_(MeCN)_4_]^4+^ and [Pd_2_ **L^4^**_2_Cl_4_]. (a) **L^4^**, comprising two sterically demanding hydrogen atoms, reacts with Pd^II^ in a 1:1 stoichiometry to form ring [Pd_2_ **L^4^**_2_(MeCN)_4_]^4+^ at room temperature in acetonitrile. Addition of 4 equiv. of chloride leads to formation of an insoluble, neutral ring [Pd_2_ **L^4^**_2_Cl_4_]. (b) ^1^H NMR spectra (600 MHz, 298 K) of ligand **L^4^** (CDCl_3_), ring [Pd_2_ **L^4^**_2_(MeCN)_4_]^4+^(0.64 mm, CD_3_CN), ring [Pd_2_ **L^4^**_2_Cl_4_] ([D_6_]DMSO) from bottom to top. (c) ESI‐HRMS of ring [Pd_2_ **L^4^**_2_(MeCN)_4_]^4+^.

Both ligands **L^2^** and **L^3^** contain one hydrogen substituent per donor group that protrudes in direction of the nitrogen bonding vector, thus creating steric congestion around the coordination site that allows construction of the [Pd_2_ **L^2/3^**_3_(MeCN)_2_]^4+^ bowl geometries. We then wondered whether the introduction of a second protruding hydrogen substituent per donor would allow to further reduce the number of ligands that can be grouped around the metal cation. Therefore, we equipped ligand **L^4^** with acridine donors on both ends in which every coordinating nitrogen is flanked by two C−H moieties (H_c_ and H_d_; Figure 3). Stirring a 1:1 mixture of sparingly soluble **L^4^** and [Pd(MeCN)_4_](BF_4_)_2_ in deuterated acetonitrile at room temperature for 1 day gave a clear yellow solution, identified as a single species [Pd_2_ **L^4^**_2_(MeCN)_4_]^4+^ by NMR spectroscopy and mass spectrometry. The ^1^H NMR spectrum showed downfield shifting of three proton signals (H_c_, H_d_, H_h_), presumably caused by metal complexation. The isotopic pattern of a prominent peak at *m*/*z* 457.6 in the high‐resolution mass spectrum is fully consistent with the simulated pattern of [Pd_2_ **L^4^**_2_(MeCN)_4_]^4+^ (Figure 3 c). Noteworthy is that the mass spectrum had to be recorded under mild ionization conditions owing to the thermal instability of ring [Pd_2_ **L^4^**_2_(MeCN)_4_]^4+^. In addition, a titration of compound [Pd_2_ **L^4^**_2_(MeCN)_4_]^4+^ with a NBu_4_Cl solution in deuterated acetonitrile further verified the postulated ring geometry, resulting in complete precipitation upon addition of four equivalents of chloride anions. The precipitate was separated, washed with chloroform and redissolved in DMSO or DMF. ^1^H NMR spectroscopy confirmed the formation of a single product, most probably neutral compound [Pd_2_ **L^4^**_2_Cl_4_], whose NMR signals were shifted with respect to free ligand **L^4^** (Figure 3 b and S24). It is interesting to note that the structural origin of the herein described ring formation is primarily based on the steric demand of the heterocyclic ligand, while analogous *trans*‐[Pd_2_ **L**_2_Cl_4_] rings have been prepared from non‐congested nitrogen ligands, chloride and Pd^II^ cations before, for example, by Puddephatt, Crowley and us.^17^ In those cases, however, the beneficial formation of uncharged complexes seemed to be the main driving force, strictly requiring anionic co‐ligands, as opposed to what we achieved herein for [Pd_2_ **L^4^**_2_(MeCN)_4_]^4+^.

**Figure 4:**
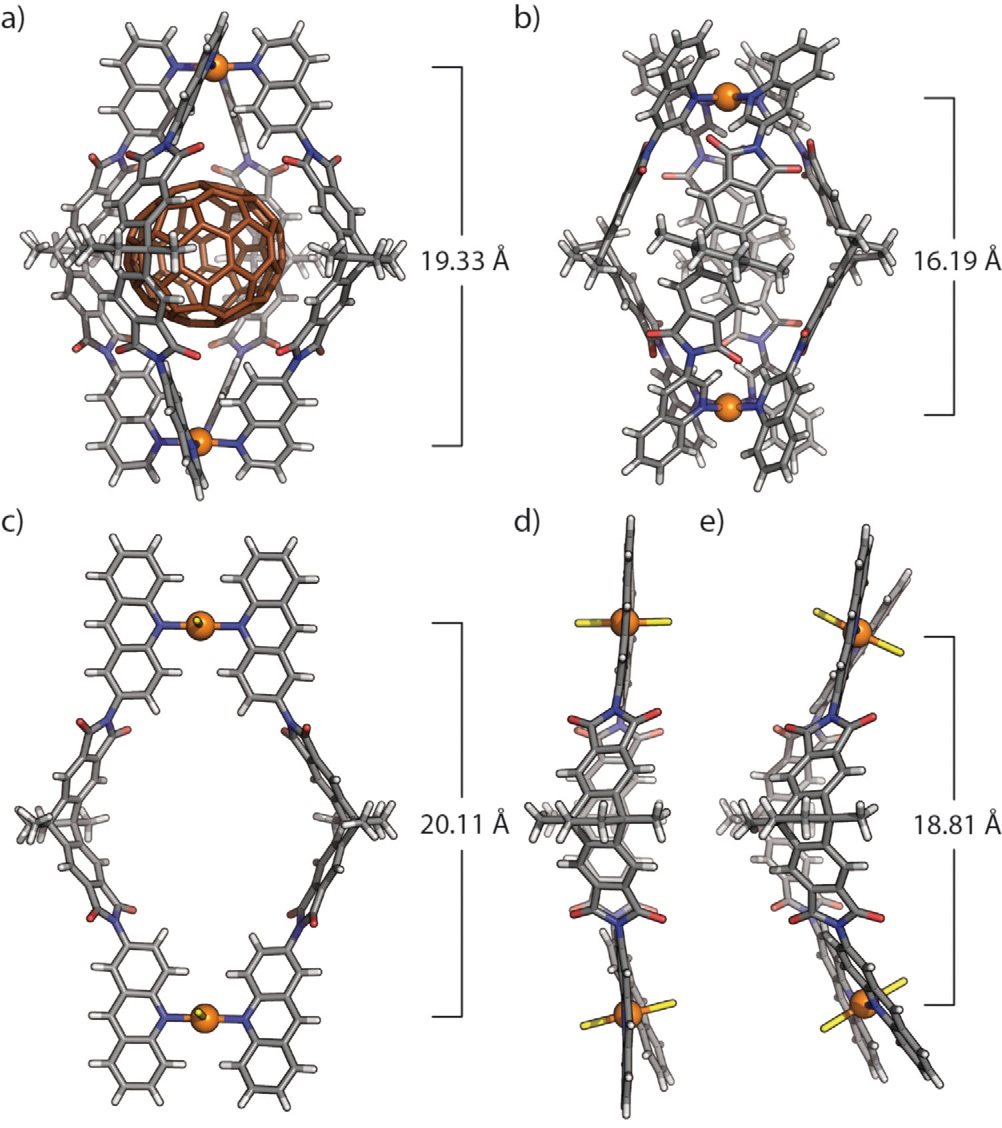
X‐ray crystal structure results. (a) [C_70_@Pd_2_ **L^2^**_4_]^4+^, (b) [Pd_2_ **L^3^**_4_]^4+^, (c) and (d) [Pd_2_ **L^4^**_2_Cl_4_], (e) side view of [Pd_2_ **L^4^**_2_Cl_4_]_B. Solvent molecules, anions and guest disorder are omitted for clarity (Pd^II^, orange; C, gray; N, blue; O, red; Cl, yellow; H, white; C_70_, brown).

Crystallization of the ring turned out to be difficult but we succeeded by adding tetrabutylammonium periodate to an acetonitrile solution of [Pd_2_ **L^4^**_2_(MeCN)_4_](BF_4_)_4_ and slow gas‐phase diffusion of benzene. Synchrotron analysis of two individual crystals obtained from the above‐mentioned conditions confirmed the *trans*‐configured ring geometries with different Pd–Pd distances of 20.11 and 18.81 Å, respectively (Figures 4 c–4 e), in line with a DFT geometry‐optimized model and the single set of proton signals observed in the ^1^H NMR spectrum. The crystallographically observed differences between the two structural polymorphs (“[Pd_2_ **L^4^**_2_Cl_4_]” and “[Pd_2_ **L^4^**_2_Cl_4_]_B”), crystallizing in two different space groups, seem to arise from the flexibility of the ring geometry in combination with different packing effects, including peripheral solvent molecules. Instead of coordinated acetonitrile molecules, we found four chloride ligands in the structures which might stem from the decomposition of CHCl_3_ or impurities. In rings [Pd_2_ **L^4^**_2_Cl_4_], the average Pd−Cl bond length of 2.33 Å observed in the crystal is close to the corresponding distance of 2.30 Å in the reported mononuclear complex Pd(acridine)_2_Cl_2_.^18^ The average distance between hydrogen atoms of opposing acridine groups is below 2.29 Å, less than double the van der Waals radius of hydrogen (1.2 Å). When fullerene binding was tested for the ring [Pd_2_ **L^4^**_2_(MeCN)_4_]^4+^ in acetonitrile, ^1^H NMR spectra exhibited broad signals assigned to inward‐pointing protons, suggesting a rather low guest loading with fast exchange between the host–guest complex and the empty ring. In addition, a color change of the solution was observed (yellow for C_60_ and orange for C_70_) and the UV/Vis spectrum showed guest‐induced absorption in the longer wavelength region (450–600 nm; Figure S31).

### Relation of structural features to guest binding

**Figure 5:**
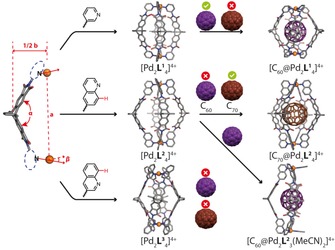
Comparison of cage family members according to their fullerene‐binding ability.

Pleasingly, a further single crystal X‐ray structure could be obtained in the course of this work, namely the host‐guest complex [C_70_@Pd_2_ **L^2^**_4_]^4+^ of the previously reported cage based on quinoline ligand **L^2^** and C_70_ fullerene (Figure 4 a).^15^ With the six structures reported in our previous work and four new structures given herein, we were able to compare the relationship between ligand chemistry, host structure and fullerene (C_60_ and C_70_) binding in a systematic and comprehensive way (Figure 5). First of all, cage [Pd_2_ **L^1^**_4_]^4+^ exhibits induced‐fit binding of only C_60_ within its cavity, while cage [Pd_2_ **L^2^**_4_]^4+^ is only capable of accommodating C_70_. With C_60_, however, the **L^2^**‐based system completely converts into bowl‐shaped host–guest complex [C_60_@Pd_2_ **L^2^**_3_(MeCN)_2_]^4+^. In contrast, cage [Pd_2_ **L^3^**_4_]^4+^ cannot bind any fullerene guest. According to X‐ray structural analysis, C_60_‐containing cage [C_60_@Pd_2_ **L^1^**_4_]^4+^, free host [Pd_2_ **L^1^**_4_]^4+^ and cage [Pd_2_ **L^3^**_4_]^4+^, having extra annelated benzene rings on both ends, show surprisingly different Pd–Pd distances from 14.61 Å over 15.94 Å to 16.19 Å, concomitant with a decrease in width (horizontal dimension orthogonal to the Pd–Pd axis; Table 1). Angle α, defined by the ligands’ benzene ring planes, varies from 122.1° over 124.3° to 126.9° at the same time. Furthermore, the donor groups can freely rotate with respect to the backbone, thus giving rise to different degrees of helical twisting of the overall cage geometry along the Pd–Pd axis. Angle β, defined as dihedral N‐Pd‐Pd‐N between the coordination bonds of one ligand with the upper and lower Pd, each, shows values of 62.8° for [C_60_@Pd_2_ **L^1^**_4_]^4+^, 1.0° for [Pd_2_ **L^1^**_4_]^4+^ and 76.9° for [Pd_2_ **L^3^**_4_]^4+^. Cavity sizes are 572 Å^3^ for [Pd_2_ **L^1^**_4_]^4+^ and 518 Å^3^ for [Pd_2_ **L^3^**_4_]^4+^.

**Table 1:** Comparison of structural details extracted from X‐ray analysis.

Structural details	[C_60_@Pd_2_ **L^1^**_4_]^4+[a]^	[Pd_2_ **L^1^**_4_]^4+^	[Pd_2_ **L^3^**_4_]^4+^	[Pd_2_ **L^2^**_4_]^4+^	[C_70_@Pd_2_ **L^2^**_4_]^4+^	[C_60_@Pd_2_ **L^2^**_3_(MeCN)_2_]^4+^
Pd–Pd distance *a* [Å]	14.61	15.94	16.19	18.80	19.33	20.22
Horizontal distance *b* [Å]^[b]^	15.12	14.12	13.15	16.90	16.17	15.14
Dihedral angle *α* [°]^[c]^	122.1	124.3	126.9	120.2	124.3	123.9
Dihedral angle *β* [°]^[d]^	62.8	1.0	76.9	1.2	1.4	0.6
Volume of cavity [Å^3^]^[e]^	780	572	518	1099	995	–

[a] Average value from three crystallographically independent cages of [C_60_@Pd_2_ **L^1^**_4_]^4+^. [b] Distance between opposite backbones as defined by the line connecting the midpoints between atoms C2 and C5. [c] Dihedral angle between the backbone's benzene planes C16_C17_C18_C22_C23_C24 and C7_C8_C9_C13_C14_C15. [d] Dihedral angle between vectors formed by coordinating N atoms and Pd atoms. [e] VOIDOO‐calculated void space with a probe radius of 3.2 Å.

A different effect was observed for host–guest complex [C_70_@Pd_2_ **L^2^**_4_]^4+^ and free cage [Pd_2_ **L^2^**_4_]^4+^ based on longer ligand **L^2^** (in terms of donor distance), where the host–guest complex shows a longer Pd–Pd distance than the free cage but therefore a shorter width. When comparing the values between the two cage families (and taking into account that C_70_ is larger than C_60_), it becomes clear that the hosts always deform in the proper direction to maximise π‐interaction to the encapsulated guests, with slightly too small [Pd_2_ **L^1^**_4_]^4+^ expanding horizontally (and shrinking along the Pd–Pd axis) to accommodate C_60_ and too large [Pd_2_ **L^2^**_4_]^4+^ shrinking horizontally—but for larger C_70_ not as much—and consequently elongating along its Pd–Pd axis. This certainly leads to the question: why does cage [Pd_2_ **L^2^**_4_]^4+^ accommodate C_70_ but not smaller C_60_ although the bowl structure based on ligand **L^2^** does bind C_60_?

Inspection of the X‐ray structures of all C_60_‐binding cages and bowls in the series reveals a horizontal width of 15.12 Å in [C_60_@Pd_2_ **L^1^**_4_]^4+^ which is remarkably similar to the corresponding distance in bowl‐shaped [C_60_@Pd_2_ **L^2^**_3_(MeCN)_2_]^4+^ (15.14 Å), thus marking the ideal horizontal width of such a host for C_60_ binding to maximize the host–guest interaction. This is further verified by the corresponding distance in the reported crystal structure of a prototypical non‐covalent adduct, that is, the co‐crystallized pair of shape‐complementary triptycene and C_60_ molecules, where the distance between the centroid of them is 7.58 Å (doubled giving 15.16 Å).^19^ If this distance, however, can only be achieved by a host under compression along the Pd–Pd axis, the structural tension that would arise might prevent guest binding at all. In case of [Pd_2_ **L^3^**_4_]^4+^, the free cage with a horizontal width of 13.15 Å would be required to widen to about 15.12 Å for the sake of binding C_60_ within the cavity. We assume that the energetic penalty for this structural change is too disadvantageous as compared with that in [Pd_2_ **L^1^**_4_]^4+^ (elongation from 14.12 Å to 15.12 Å). In other words, fullerene binding is only observed within the cage when the attractive π–π and CH–π host–guest interactions can overcome any binding‐induced energetic disadvantage.

Likewise, the above‐mentioned non‐existence of species [C_60_@Pd_2_ **L^2^**_4_]^4+^ can also be explained by this hypothesis: the favorable host–guest interaction is not strong enough to conquer the unfavorable structural strain in a compressed cage. Hence, the system escapes this dilemma by releasing one ligand, yielding bowl geometry [C_60_@Pd_2_ **L^2^**_3_(MeCN)_2_]^4+^ that does not suffer corresponding strain due to its less structurally constrained metal sites. In C_70_ binding, however, the attractive host–guest interaction is larger due to C_70_′s larger surface area. In addition, its larger diameter means lower requirements for the cage to shrink in the horizontal direction. This can be observed when comparing the crystal structures of [Pd_2_ **L^2^**_4_]^4+^ and [C_70_@Pd_2_ **L^2^**_4_]^4+^, where the horizontal distance of the cage has been only reduced from 16.90 Å to 16.17 Å upon binding C_70_ instead of having to reach a much more demanding 15.14 Å as would be required for binding C_60_. This hypothesis could be further confirmed by analyzing the guest position in the crystal structure of [C_70_@Pd_2_ **L^2^**_4_]^4+^, showing that the longest axis of the ellipsoidal C_70_, which is 1.0 Å longer than the diameter of spherical C_60_, is clearly located (but uniformly disordered) in the equatorial plane of the inner cavity (Figure S32).^20^

## Conclusions

In this work, we describe the expansion of a family of bis‐monodentate ligands based on a curved backbone that allows corresponding metallo‐supramolecular hosts to bind fullerenes in their interior. While pyridine‐based ligands form symmetric [Pd_2_L_4_] cages, sterically more demanding quinoline‐based systems lead to the formation of bowl‐shaped structures when a Pd^II^:ligand ratio of 2:3 is adjusted. Even bulkier acridine‐based ligands lead to the formation of rings with two acridine donors per square planar palladium center whose remaining coordination sites are either occupied by solvent or halide molecules. We systematically studied structural transformation and the uptake of C_60_ and C_70_ fullerene guests by NMR spectroscopic, mass spectrometric and X‐ray diffraction methods. The large amount of structural data allowed us to win detailed insight into the factors governing guest binding and selectivity. Together, the herein reported findings add substantial understanding to the structure‐function relationship in fullerene‐binding self‐assembled hosts, thereby helping to construct further hosts to solubilize carbon materials, selective recognition systems and nano‐confined reaction environments.

## Experimental Section

The detailed synthesis and characterization of all the compounds are described in the Supporting Information. CCDC https://www.ccdc.cam.ac.uk/services/structures?id=doi:10.1002/chem.201903317 contain the supplementary crystallographic data for this paper. These data are provided free of charge by http://www.ccdc.cam.ac.uk/ and Fachinformationszentrum Karlsruhe Access Structures service.

## Conflict of interest

The authors declare no conflict of interest.

## Supporting information

As a service to our authors and readers, this journal provides supporting information supplied by the authors. Such materials are peer reviewed and may be re‐organized for online delivery, but are not copy‐edited or typeset. Technical support issues arising from supporting information (other than missing files) should be addressed to the authors.

Supplementary
