# Supplementary material for: Tunable Fullerene Affinity of Cages, Bowls and Rings Assembled by PdII Coordination Sphere Engineering
Source: Chemistry. 2019 Oct 24;25(65):14921–7. doi: 10.1002/chem.201903317 (PMC6899814; doi:10.1002/chem.201903317)
Supplement: Supplementary file 1 — Supplementary [file CHEM-25-14921-s001.pdf]

CHEMISTRY
A European Journal

Supporting Information

Tunable Fullerene Affinity of Cages, Bowls and Rings Assembled by Pd^{II} Coordination Sphere Engineering

Bin Chen,[a] Shinnosuke Horiuchi,[a, b] Julian J. Holstein,[a] Jacopo Tessarolo,[a] and Guido H. Clever*[a]

chem_201903317_sm_miscellaneous_information.pdf

Contents:

1 Materials and methods **2**

1.1 Materials *2*

1.2 Analytical techniques *3*

2 Synthesis of ligands L^3 and L^4 **4**

2.1 Synthesis of ligand L^3 *4*

2.2 Synthesis of ligand L^4 *5*

3 Formation and characterization of metallosupramolecular assemblies **7**

3.1 Bowl $[Pd_2L^3_3(MeCN)_2]^{4+}$ *7*

3.2 Cage $[Pd_2L^3_4]^{4+}$ *12*

3.3 Ring $[Pd_2L^4_2(MeCN)_4]^{4+}$ *14*

3.4 Titration of ring $[Pd_2L^4_2(MeCN)_4]^{4+}$ with chloride anions *17*

3.5 Ring $[Pd_2L^4_2Cl_4]$ *17*

4 Fullerene binding studies **20**

4.1 Fullerene binding experiment with cage $[Pd_2L^3_4]^{4+}$ *20*

4.2 Fullerene binding experiment with ring $[Pd_2L^4_2(MeCN)_4]^{4+}$ *22*

5 X-Ray data **23**

5.1 General methods *23*

5.2 Crystal structure of $[C_{70}@Pd_2L^2_4](BF_4)_4$ *25*

5.3 Crystal structure of $[Pd_2L^3_4](BF_4)_4$ *27*

5.4 Crystal structure of $[Pd_2L^4_2Cl_4]$ *28*

5.5 Crystal structure of $[Pd_2L^4_2Cl_4]$_B *31*

5.6 Calculation of cavity volumes *32*

6 Computational studies **33**

6.1 DFT calculation of the energy change during the conversion from $[Pd_2L^2_3(MeCN)_2]^{4+}$ to $[Pd_2L^2_4]^{4+}$ *34*

6.2 DFT calculation of the energy change during the conversion from $[Pd_2L^3_3(MeCN)_2]^{4+}$ to $[Pd_2L^3_4]^{4+}$ *35*

6.3 Comparison of the DFT minimized energies of cis-$[Pd_2L^4_2(MeCN)_4]^{4+}$ and trans-$[Pd_2L^4_2(MeCN)_4]^{4+}$ *36*

7 References **36**

1 Materials and methods

1.1 Materials

All chemicals were obtained from commercial sources and used without further purification. Fullerenes C_{60} and C_{70} were purchased from ABCR with a purity of 99.95% and Sigma-Aldrich with a purity of 98%, respectively.

Syntheses and characterization of ligands $\mathbf{L^1}$ and $\mathbf{L^2}$ as well as their self-assembled cages and bowls, i.e. $[Pd_2\mathbf{L^1}_4]^{4+}$, $[C_{60}@Pd_2\mathbf{L^1}_4]^{4+}$, $[Pd_2\mathbf{L^2}_3(MeCN)_2]^{4+}$, $[Pd_2\mathbf{L^2}_4]^{4+}$, $[C_{60}@Pd_2\mathbf{L^2}_3(MeCN)_2]^{4+}$, $[C_{70}@Pd_2\mathbf{L^2}_3(MeCN)_2]^{4+}$ and $[C_{70}@Pd_2\mathbf{L^2}_4]^{4+}$ have been reported previously.[1]

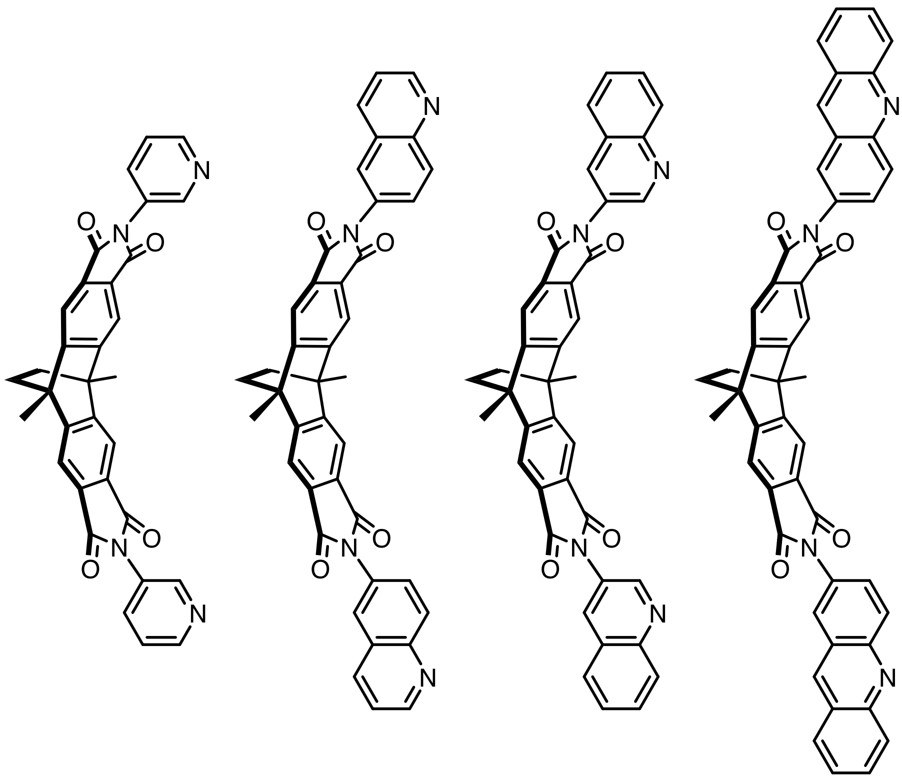

$\mathbf{L^1}$ $\mathbf{L^2}$ $\mathbf{L^3}$ $\mathbf{L^4}$

Figure S1 Chemical structures of all ligands used in this study.

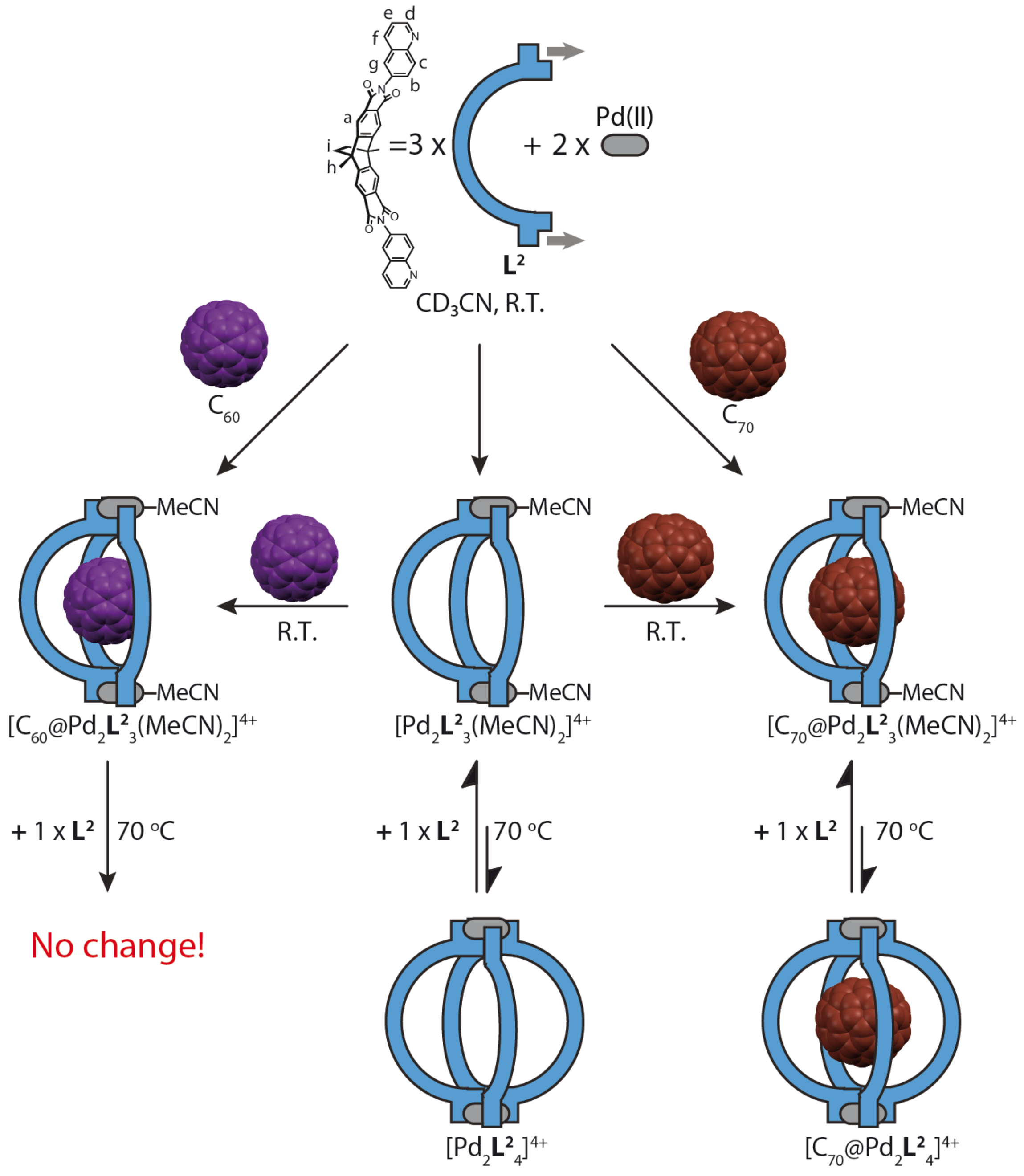

Figure S2 Overview of previously reported self-assembly and host-guest results.[1]

1.2 Analytical techniques

Gel permeation chromatography (GPC) purification of ligands was performed on a JASCO LC-9210 II NEXT running with $CHCl_3$ (HPLC grade) containing 0.5% (v/v) triethylamine. NMR measurements were all conducted at 298 K on Avance-500 and Avance-600 instruments from Bruker and an INOVA 500 MHz machine from Varian. Chemical shifts for ^{1}H and ^{13}C are reported in ppm on the δ scale; ^{1}H and ^{13}C signals were referenced to the residual solvent peak: acetonitrile (1.94 ppm, 1.32 ppm); chloroform (7.26 ppm, 77.16 ppm) or dimethyl sulfoxide (2.50 ppm, 39.52 ppm). The following abbreviations are used to describe signal multiplicity for ^{1}H NMR spectra: s: singlet, d: doublet, t: triplet, dd: doublet of doublets; dt: doublet of triplets; m: multiplet, br: broad. All proton signals of supramolecular cage or bowl compounds were assigned

with the aid of 2D NMR spectra. The proton signal at 7.58 ppm arising in some NMR spectra (298 K, CD3CN) can be assigned to traces of residual $CHCl_3$, co-crystallized with some ligands after purification. High resolution electrospray ionization mass spectrometry (ESI HRMS) was performed on Bruker Apex IV FTICR, Bruker compact and Bruker timsTOF ESI mass spectrometers. The samples were diluted with spectrum-grade CH_3CN (1:10) prior to the measurement. UV-Vis spectra were recorded on an Agilent DAD HP-8453 UV-Vis spectrophotometer using quartz cuvettes with an optical path length of 1 mm.

2 Synthesis of ligands L^3 and L^4

Ligands **L^3** and **L^4** were prepared from reported bis-anhydride (9,10-dimethyl-9,10-dihydro-9,10-ethanoanthracene-2,3,6,7-dianhydride)[2] and the corresponding powdered aromatic amines under nitrogen atmosphere as described below.

2.1 Synthesis of ligand L^3

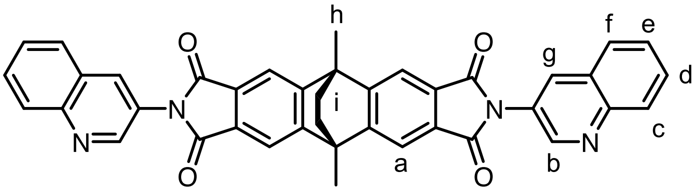

L^3

Under a nitrogen atmosphere, ligand **L^3** was prepared from reported bis-anhydride (9,10-dimethyl-9,10-dihydro-9,10-ethanoanthracene-2,3,6,7-dianhydride) (93.6 mg, 0.25 mmol, 1 eq.) and powdered 3-aminoquinoline (720.7 mg, 5.00 mmol, 20 eq.) by heating the mixture of solids without solvent in a preheated oil bath to 165 °C for 10 min. After the black melt cooled to room temperature, it was taken up into 5 mL chloroform, sonificated and the suspension was immediately subjected to flash column chromatography on silica gel ($CHCl_3$: MeOH = 50 : 1) to give the crude product. This was further purified via recycling gel permeation chromatography and the solvent was removed under reduced pressure to yield the desired product as a white powder (100.1 mg, 64 %).

^{1}H NMR (600 MHz, 298 K, $CDCl_3$): δ (ppm) = 9.02 (d, J = 2.4 Hz, 2H), 8.28 (d, J = 2.4 Hz, 2H), 8.17 (d, J = 8.4 Hz, 2H), 7.99 (s, 4H), 7.90 (d, J = 8.1 Hz, 2H), 7.78 (m, 2H), 7.62 (m, 2H), 2.22 (s, 6H), 1.81 (s, 4H).

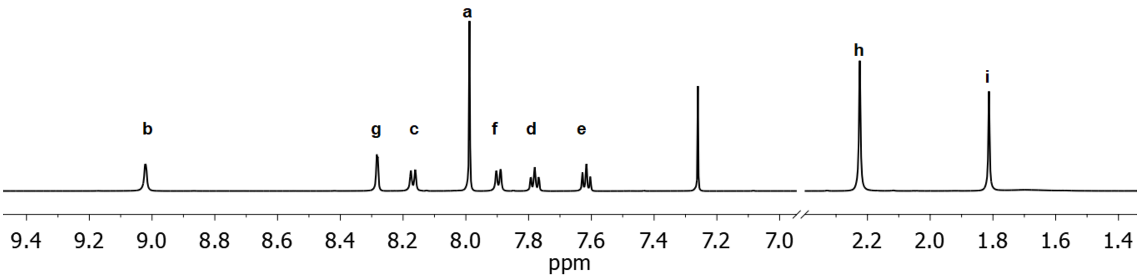

Figure S3 ^{1}H NMR spectrum (600 MHz, 298 K, $CDCl_3$) of **L^3**.

^{13}C NMR (151 MHz, 298 K, $CDCl_3$): δ (ppm) = 167.28, 152.84, 147.99, 147.02, 132.63, 130.31, 130.17, 129.52, 128.20, 127.76, 127.53, 125.74, 116.96, 44.39, 35.00, 18.86.

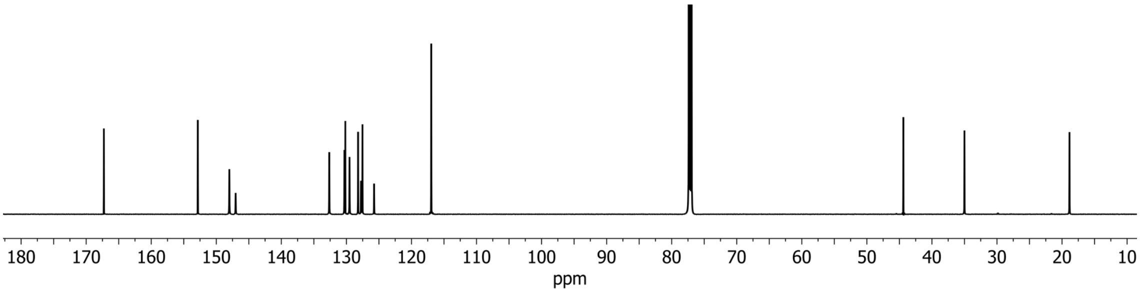

Figure S4 ^{13}C NMR spectrum (151 MHz, 298 K, $CDCl_3$) of **L^3**.

ESI HRMS ($C_{40}H_{26}N_4O_4$): $[M + H]^+$ calcd. for $C_{40}H_{27}N_4O_4$ 627.2027; found 627.2015.

2.2 Synthesis of ligand L^4

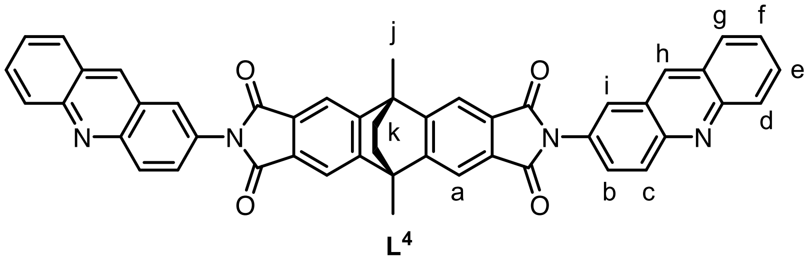

L^4

Under a nitrogen atmosphere, ligand **L^4** was prepared from reported bis-anhydride (9,10-dimethyl-9,10-dihydro-9,10-ethanoanthracene-2,3,6,7-dianhydride) (37.4 mg, 0.10 mmol, 1 eq.) and powdered 2-aminoacridine (194.2 mg, 1.00 mmol, 10 eq.) by heating the mixture of solids without solvent in a preheated oil bath to 230 °C for 10 min. After the black melt cooled to room temperature, it was taken up into 10 mL chloroform, sonificated and the suspension was immediately subjected to flash column chromatography on silica gel ($CHCl_3$: MeOH = 30 : 1) to give the crude product. This was further purified via recycling gel permeation chromatography and the solvent was removed under reduced pressure to yield the desired product as a pale-yellow powder (41.6 mg, 57 %).

^{1}H NMR (700 MHz, 298 K, $CDCl_3$): δ (ppm) = 8.85 (s, 2H), 8.36 (d, J = 9.3 Hz, 2H), 8.27 (d, J = 8.8 Hz, 2H), 8.14 (d, J = 2.3 Hz, 2H), 8.03 (d, J = 8.4 Hz, 2H), 8.00 (s, 4H), 7.88 (dd, J = 9.3, 2.3 Hz, 2H), 7.83 (m, 2H), 7.58 (m, 2H), 2.25 (s, 6H), 1.83 (s, 4H).

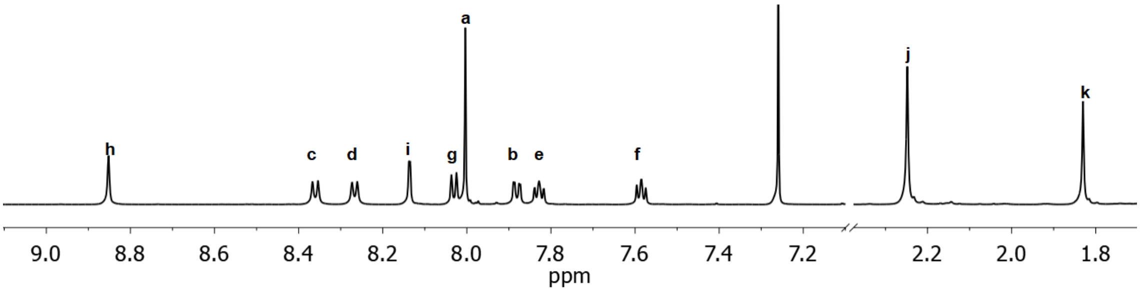

Figure S5 ^{1}H NMR spectrum (700 MHz, 298 K, $CDCl_3$) of **L^4**.

^{13}C NMR (151 MHz, 298 K, $CDCl_3$): δ (ppm) = 167.56, 152.80, 149.69, 147.91, 136.69, 130.93, 130.83, 130.25, 129.72, 129.13, 128.69, 128.36, 126.92, 126.40, 126.21, 125.20, 116.90, 44.40, 35.05, 18.94.

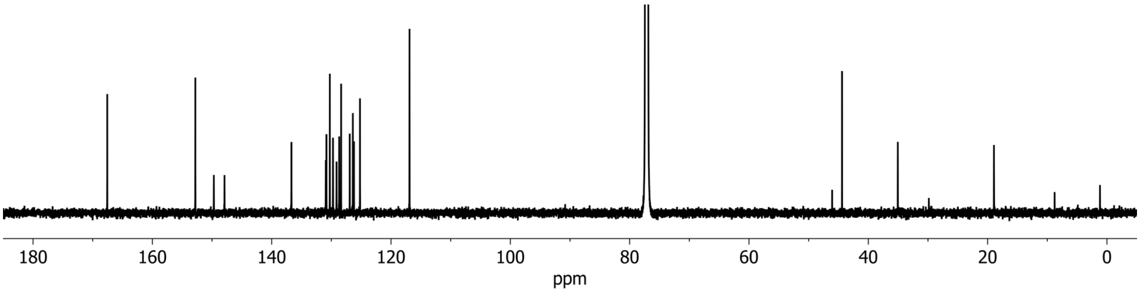

Figure S6 ^{13}C NMR spectrum (151 MHz, 298 K, $CDCl_3$) of **L^4**.

ESI HRMS ($C_{48}H_{30}N_4O_4$): $[M + H]^+$ calcd. for $C_{48}H_{31}N_4O_4$ 727.2340; found 727.2445; $[M + 2H]^{2+}$ calcd. for $C_{48}H_{32}N_4O_4$ 364.1206; found 364.1260.

3 Formation and characterization of metallosupramolecular assemblies

3.1 Bowl $[Pd_2L^3_3(MeCN)_2]^{4+}$

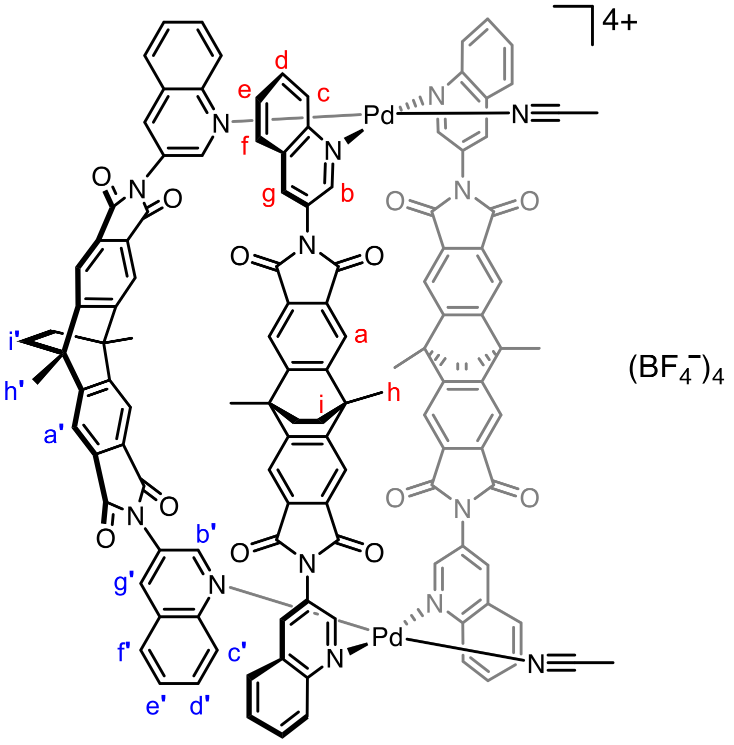

$[Pd_2\mathbf{L}^3_3(MeCN)_2]^{4+}$

A solution of $[Pd(MeCN)_4](BF_4)_2$ (114.3 µL, 15 mM/CD_3CN, 1.71 µmol, 1 eq.) was combined with ligand $\mathbf{L^3}$ (1.6 mg, 2.57 µmol, 1.5 eq.) in CD_3CN (1224 µL) and stirred at room temperature for 2 h to give a 0.64 mM solution of bowl $[Pd_2\mathbf{L}^3_2(MeCN)_2]^{4+}$. NMR characterization was performed right after preparation of a fresh sample.

^{1}H NMR (600 MHz, 298 K, CD_3CN): δ (ppm) = 10.20 (d, J = 2.1 Hz, 4H), 9.97 (d, J = 2.1 Hz, 2H), 9.78 (d, J = 8.6 Hz, 4H), 9.49 (d, J = 8.8 Hz, 2H), 8.77 (d, J = 2.1 Hz, 4H), 8.68 (d, J = 2.0 Hz, 2H), 8.35 (m, 4H), 8.15 (dd, J = 8.3, 1.3 Hz, 4H), 8.04 (s, 4H), 7.92 (m, 12H), 7.79 (s, 4H), 7.63 (m, 2H), 2.23 (s, 6H), 2.20 (s, 6H), 2.15 (s, mixed with water peak in CD_3CN), 1.87 (s, 4H), 1.76 – 1.72 (m, 4H), 1.71 – 1.67 (m, 4H).

A signal at 2.15 ppm overlapping with the solvent residual peak in the aliphatic region could be assigned via 2D NMR spectroscopy.

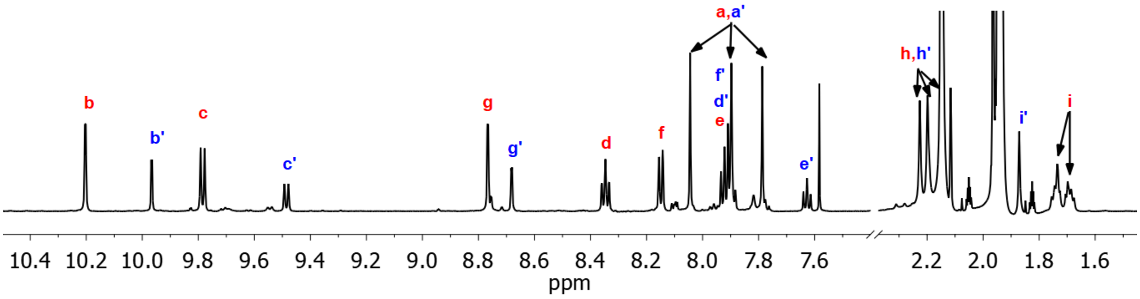

Figure S7 ^{1}H NMR spectrum (600 MHz, 298 K, CD_3CN) of $[Pd_2\mathbf{L^3}_3(MeCN)_2]^{4+}$.

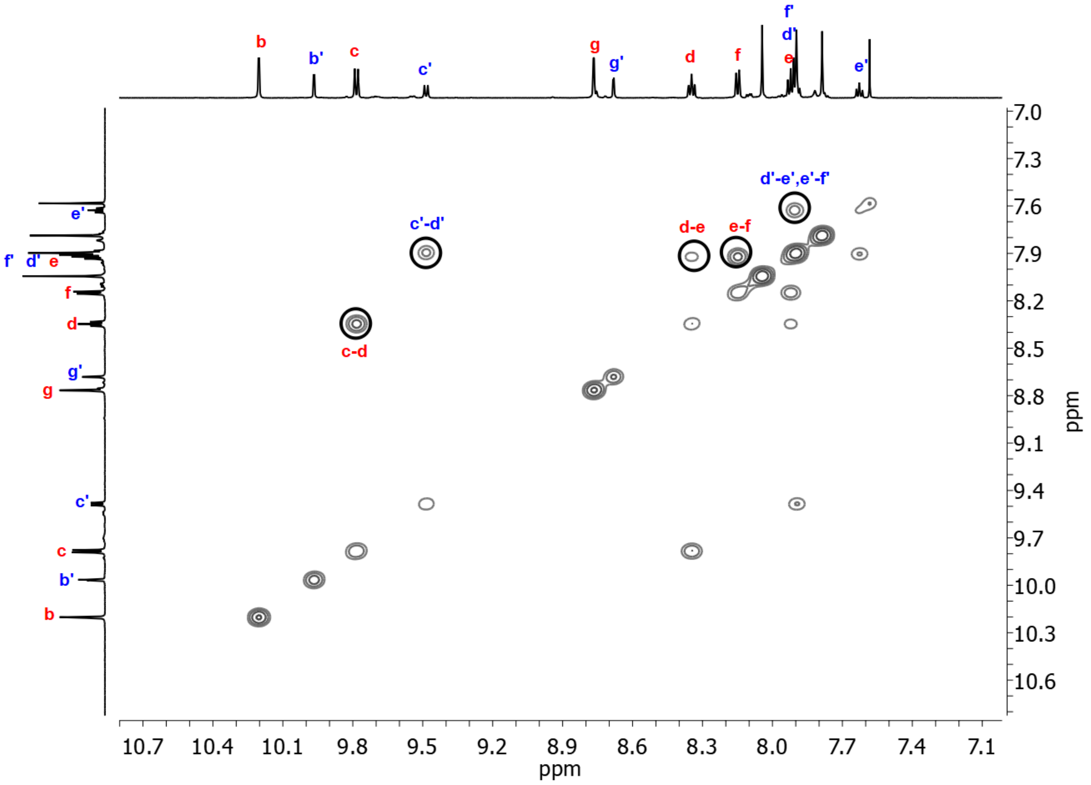

Figure S8 Partial ^{1}H – ^{1}H COSY spectrum (600 MHz, 298 K, CD_3CN) of $[Pd_2\mathbf{L^3}_3(MeCN)_2]^{4+}$.

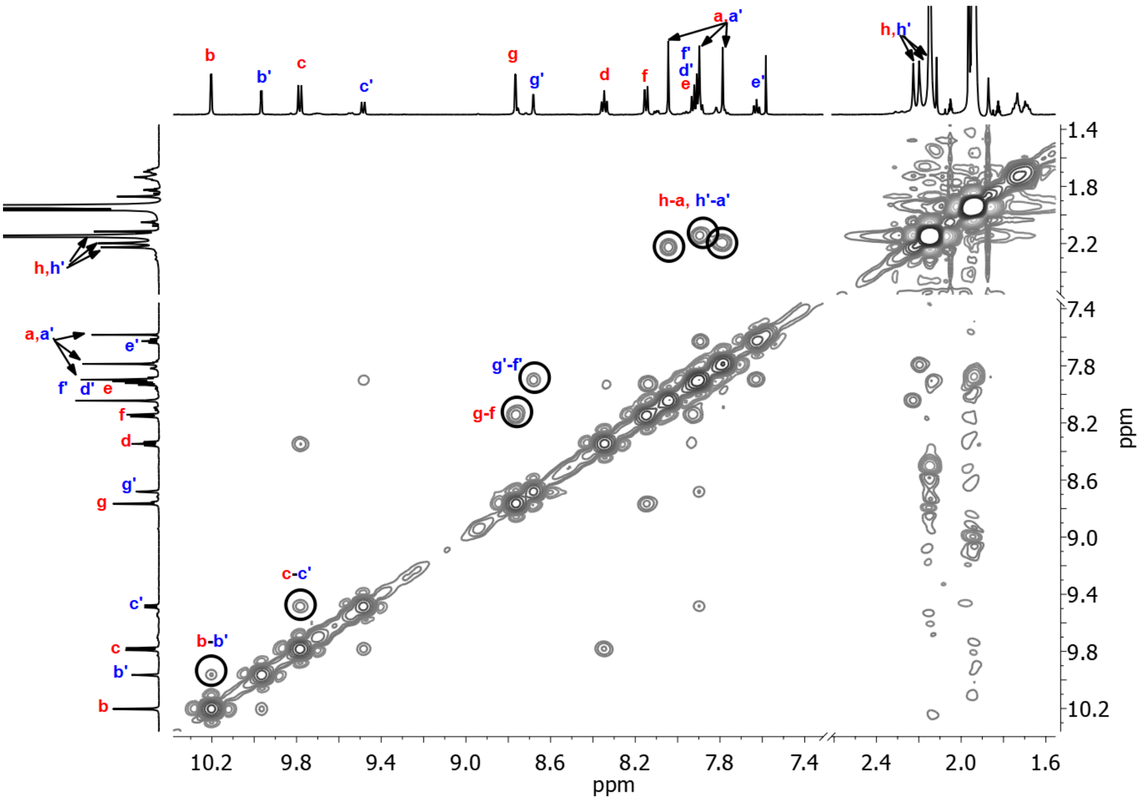

Figure S9 Partial ^{1}H – ^{1}H NOESY spectrum (600 MHz, 298 K, CD_3CN) of $[Pd_2\mathbf{L^3}_3(MeCN)_2]^{4+}$.

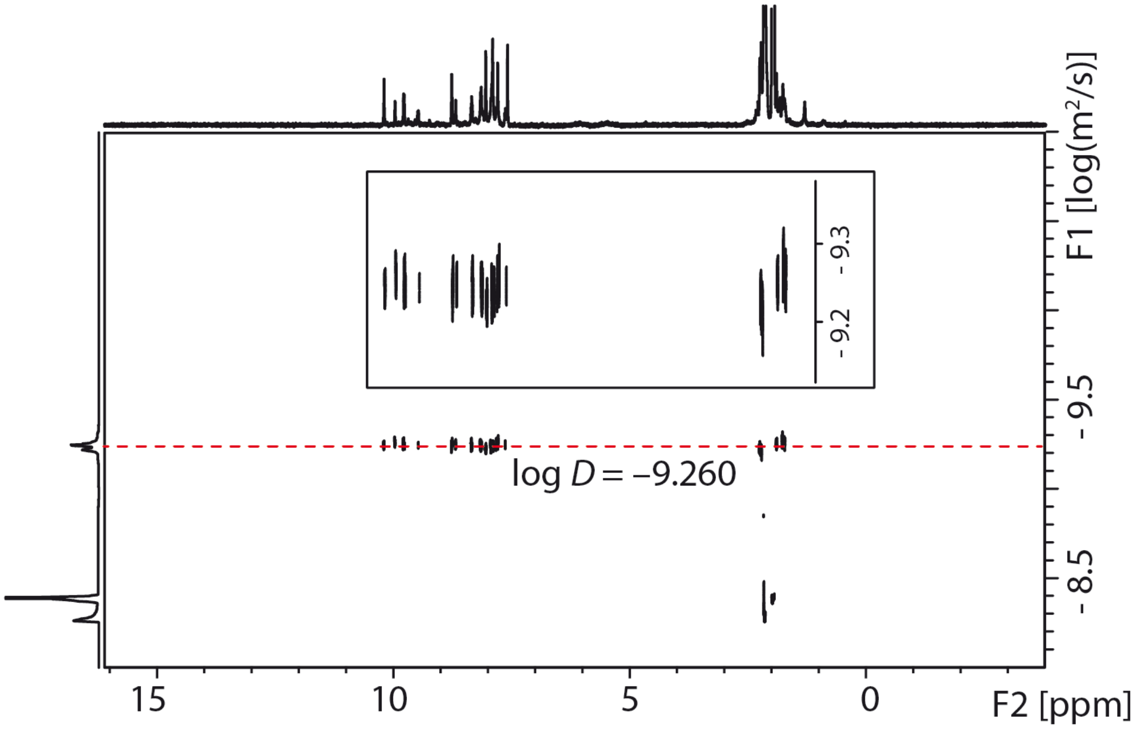

Figure S10 DOSY spectrum (500 MHz, 298 K, CD_3CN) of $[Pd_2\mathbf{L^3}_3(MeCN)_2]^{4+}$: diffusion coefficient = 5.5 x 10^{-10} m^2s^{-1}, log D = −9.26, r = 11.5 Å.

ESI HRMS ($C_{124}H_{84}N_{14}O_{12}Pd_2B_4F_{16}$): $[Pd_2\mathbf{L^3}_3(MeCN)_2]^{4+}$ calcd. for $C_{124}H_{84}N_{14}O_{12}Pd_2$ 543.6123; found 543.6161; $[Pd_2\mathbf{L^3}_3+BF_4]^{3+}$ calcd. for $C_{120}H_{78}N_{12}O_{12}Pd_2BF_4$ 726.1335; found 726.1458; $[Pd_2\mathbf{L^3}_3+2BF_4]^{2+}$ calcd. for $C_{120}H_{78}N_{12}O_{12}Pd_2B_2F_8$ 1132.7023; found 1132.7227.

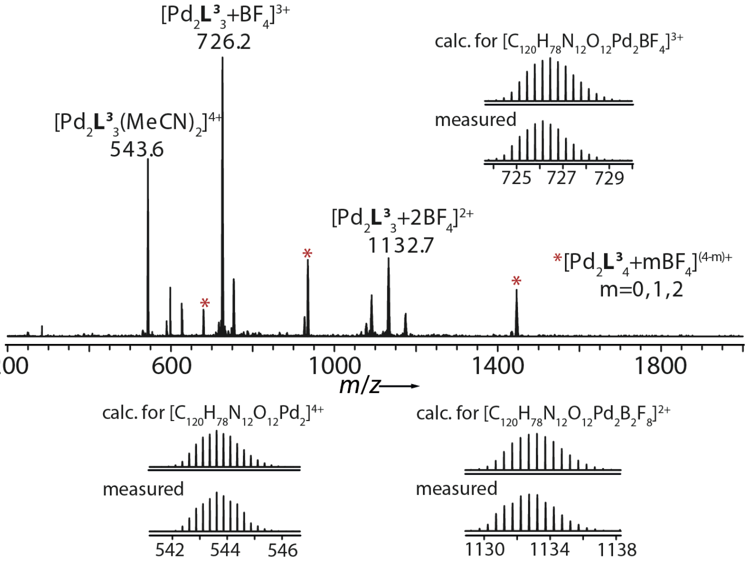

Figure S11 ESI mass spectrum of $[Pd_2\mathbf{L^3}_3(MeCN)_2]^{4+}$. *The observed $[Pd_2\mathbf{L^3}_4]^{4+}$ species is caused by partial structural reorganization of the thermodynamically unstable species $[Pd_2\mathbf{L^3}_3(MeCN)_2]^{4+}$ under the measurement conditions.

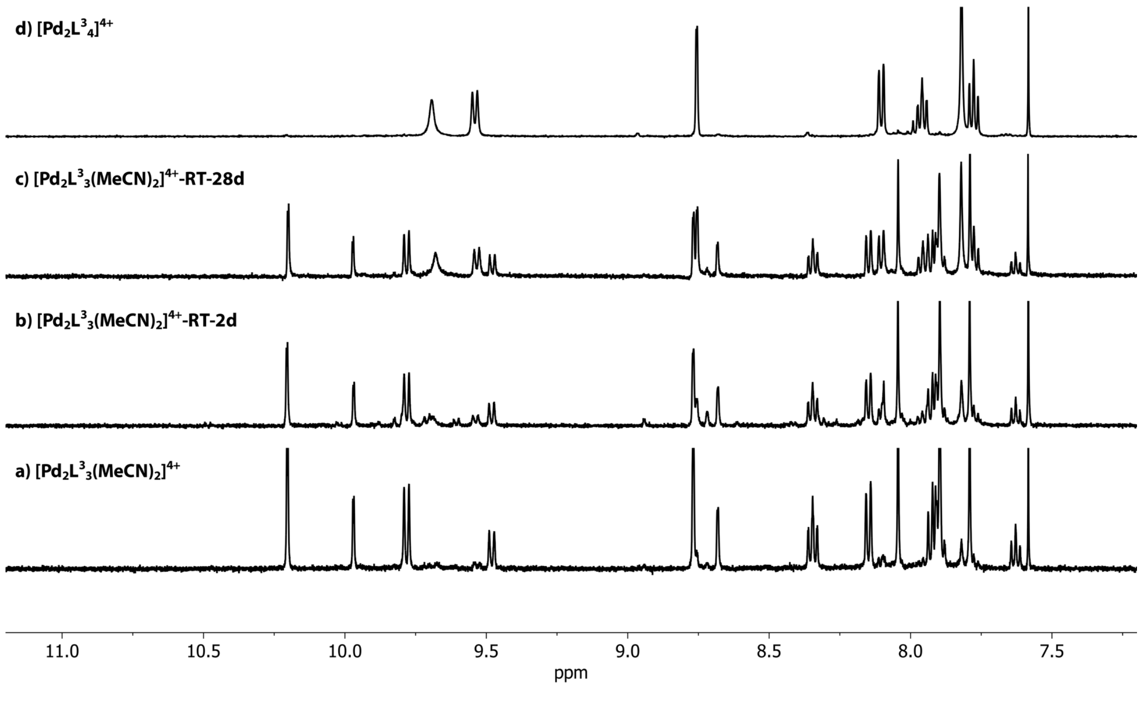

Figure S12 ^{1}H NMR spectra (500 MHz, 298 K, CD_3CN) of (a) freshly prepared $[Pd_2\mathbf{L^3}_3(MeCN)_2]^{4+}$ (0.64 mM), (b) and (c) partial conversion of $[Pd_2\mathbf{L^3}_3(MeCN)_2]^{4+}$ to $[Pd_2\mathbf{L^3}_4]^{4+}$ after standing for 2 d or 28 d at room temperature, indicating the instability of bowl $[Pd_2\mathbf{L^3}_3(MeCN)_2]^{4+}$, (d) pure $[Pd_2\mathbf{L^3}_4]^{4+}$ (0.64 mM).

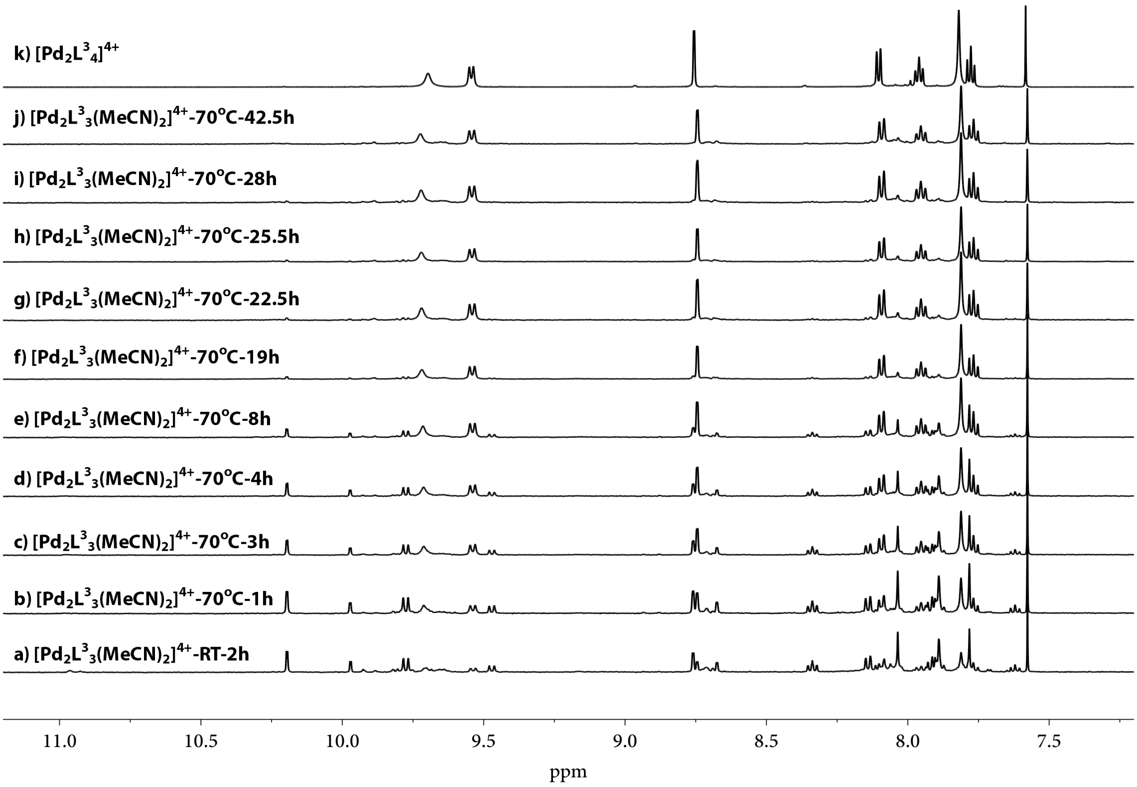

Figure S13 ^{1}H NMR spectroscopic monitoring of the conversion of freshly prepared $[Pd_2\mathbf{L^3}_3(MeCN)_2]^{4+}$ (0.64 mM) at 70 °C into $[Pd_2\mathbf{L^3}_4]^{4+}$ after 1 day (500 MHz, CD_3CN).

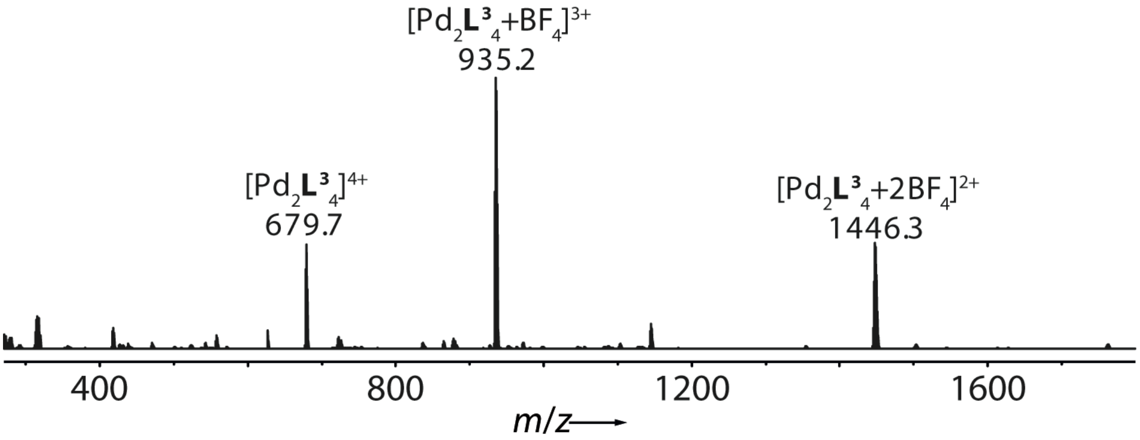

Figure S14 ESI mass spectrum of the resulting solution when freshly prepared $[Pd_2\mathbf{L^3}_3(MeCN)_2]^{4+}$ was heated at 70 °C for 28 h, showing a complete reorganization into cage $[Pd_2\mathbf{L^3}_4]^{4+}$.

3.2 Cage $[Pd_2L^3{}_4]^{4+}$

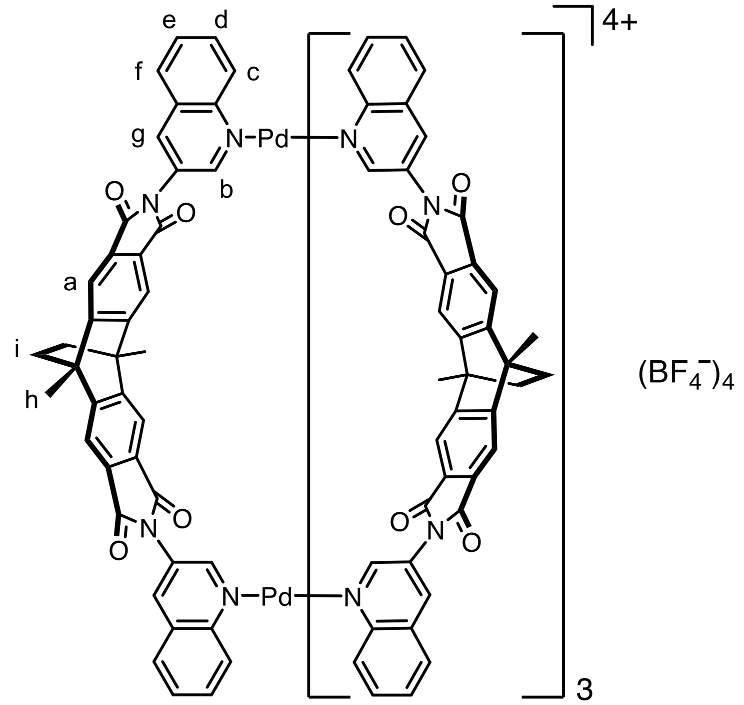

A solution of $[Pd(MeCN)_4](BF_4)_2$ (638.8 µL, 15 mM/CD_3CN, 9.58 µmol, 1 eq.) was combined with ligand **L³** (12.0 mg, 19.16 µmol, 2 eq.) in CD_3CN (6844 µL) and heated at 70 °C for 2 d to give a 0.64 mM solution of cage $[Pd_2L^3{}_4]^{4+}$.

¹H NMR (600 MHz, 298 K, CD_3CN): *δ* (ppm) = 9.70 (s, 8H), 9.54 (d, J = 8.7 Hz, 8H), 8.76 (d, J = 2.0 Hz, 8H), 8.10 (dd, J = 8.3, 1.3 Hz, 8H), 7.96 (m, 8H), 7.82 (s, 16H), 7.78 (m, 8H), 2.19 (s, 24H), 1.74 (s, 16H).

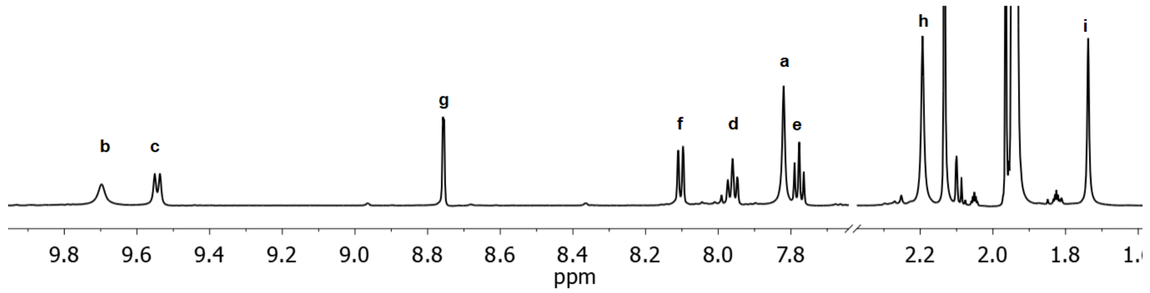

Figure S15 ¹H NMR spectrum (600 MHz, 298 K, CD_3CN) of $[Pd_2L^3{}_4]^{4+}$.

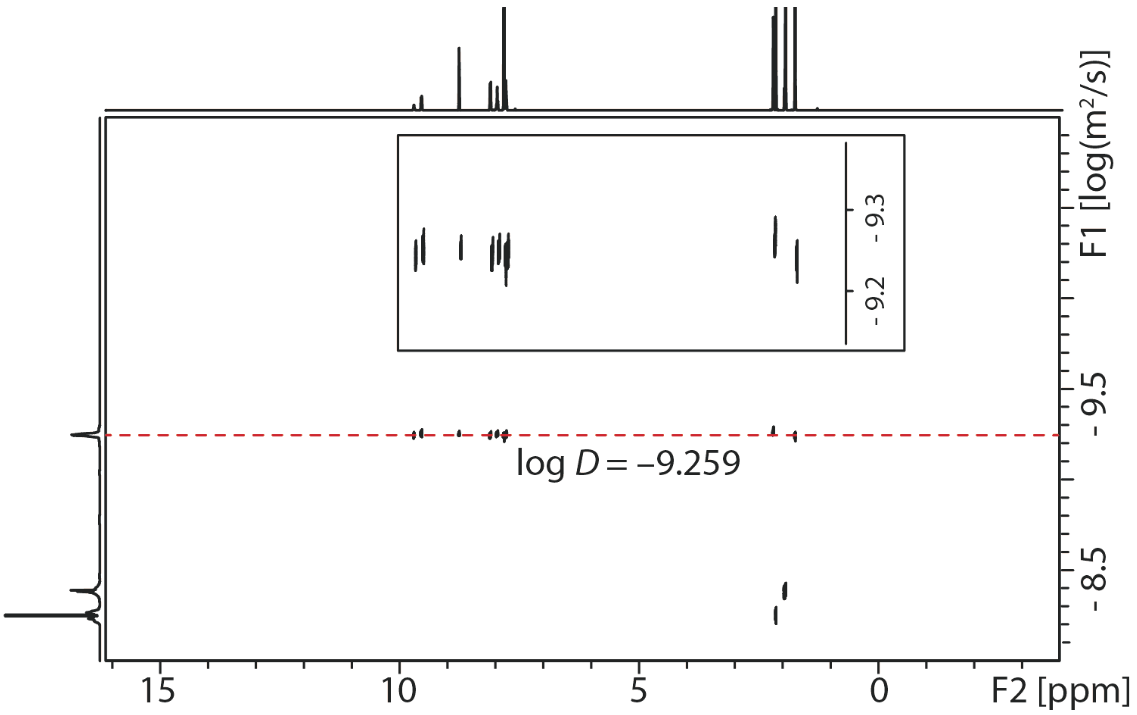

Figure S16 DOSY spectrum (500 MHz, 298 K, CD_3CN) of $[Pd_2\mathbf{L^3}_4]^{4+}$: diffusion coefficient = 5.5 x 10^{-10} m^2s^{-1}, log *D* = −9.26, r = 11.5 Å.

ESI HRMS ($C_{160}H_{104}N_{16}O_{16}Pd_2B_4F_{16}$): $[Pd_2\mathbf{L^3}_4]^{4+}$ calcd. for $C_{160}H_{104}N_{16}O_{16}Pd_2$ 679.6482; found 679.6539; $[Pd_2\mathbf{L^3}_4+BF_4]^{3+}$ calcd. for $C_{160}H_{104}N_{16}O_{16}Pd_2BF_4$ 935.1990; found 935.2072; $[Pd_2\mathbf{L^3}_4+2BF_4]^{2+}$ calcd. for $C_{160}H_{104}N_{16}O_{16}B_2F_8$ 1446.3007; found 1446.3137.

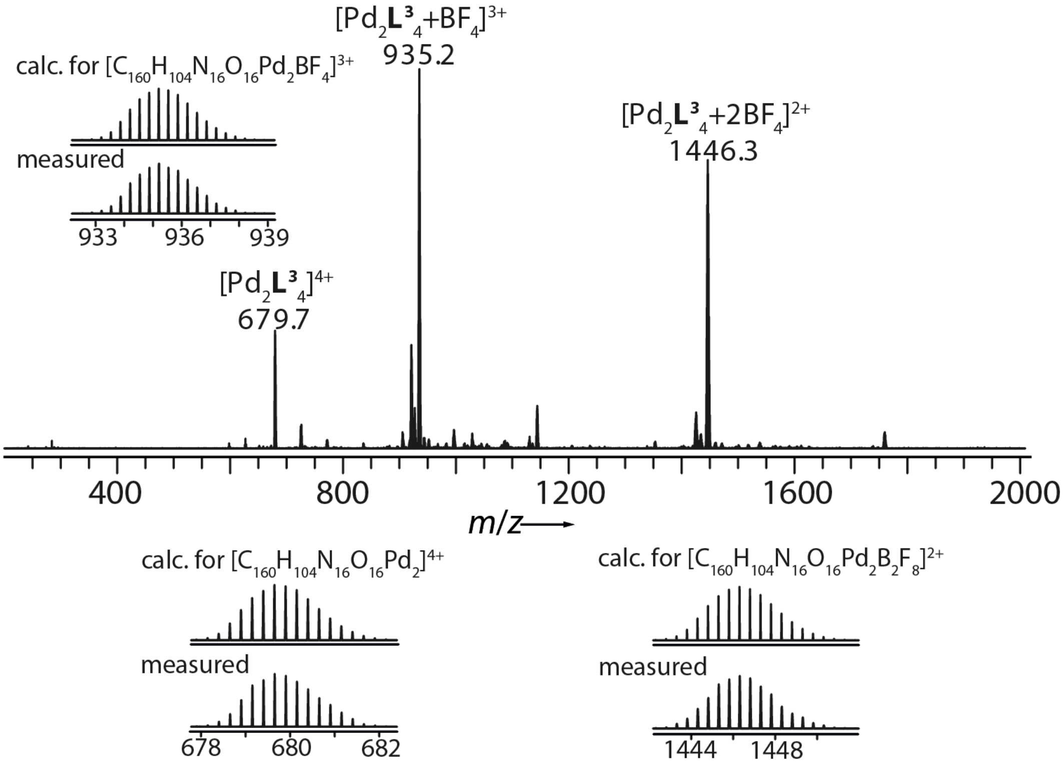

Figure S17 ESI mass spectrum of $[Pd_2\mathbf{L^3}_4]^{4+}$.

3.3 Ring $[Pd_2L^4_2(MeCN)_4]^{4+}$

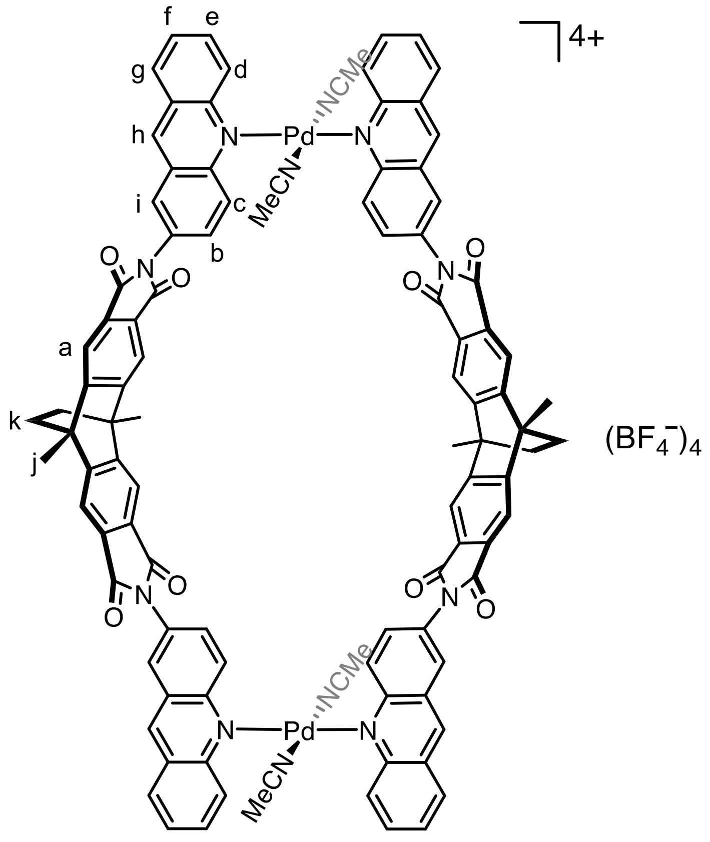

$[Pd_2\mathbf{L}^4_2(MeCN)_4]^{4+}$

A solution of $[Pd(MeCN)_4](BF_4)_2$ (162.4 µL, 15 mM/CD_3CN, 2.44 µmol, 1 eq.) was combined with ligand $\mathbf{L}^4$ (1.8 mg, 2.44 µmol, 1 eq.) in CD_3CN (1740 µL) and stirred at room temperature for 1 d to give a 0.64 mM solution of ring $[Pd_2\mathbf{L}^4_2(MeCN)_4]^{4+}$.

^{1}H NMR (600 MHz, 298 K, CD_3CN): δ (ppm) = 10.30 (d, J = 9.4 Hz, 4H), 10.27 (d, J = 8.9 Hz, 4H), 9.70 (s, 4H), 8.67 (m, 4H), 8.57 (dd, J = 9.3, 2.3 Hz, 4H), 8.52 (d, J = 2.2 Hz, 4H), 8.47 (d, J = 8.3 Hz, 4H), 8.08 – 8.05 (m, 4H), 8.03 (s, 8H), 2.30 (s, 12H), 1.89 (s, 8H).

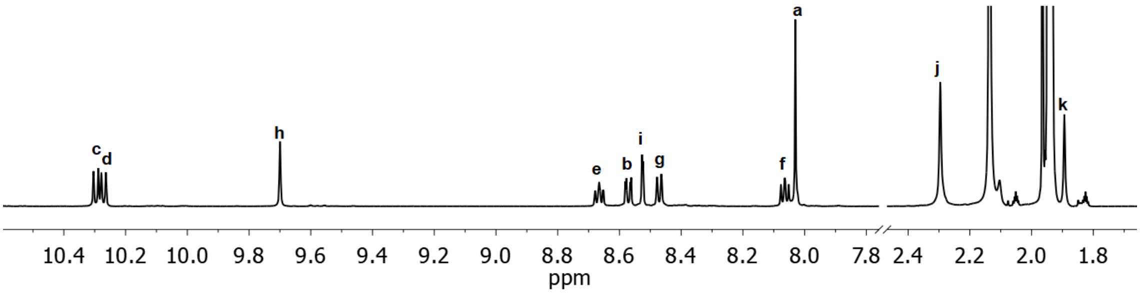

Figure S18 ^{1}H NMR spectrum (600 MHz, 298 K, CD_3CN) of $[Pd_2\mathbf{L}^4_2(MeCN)_4]^{4+}$.

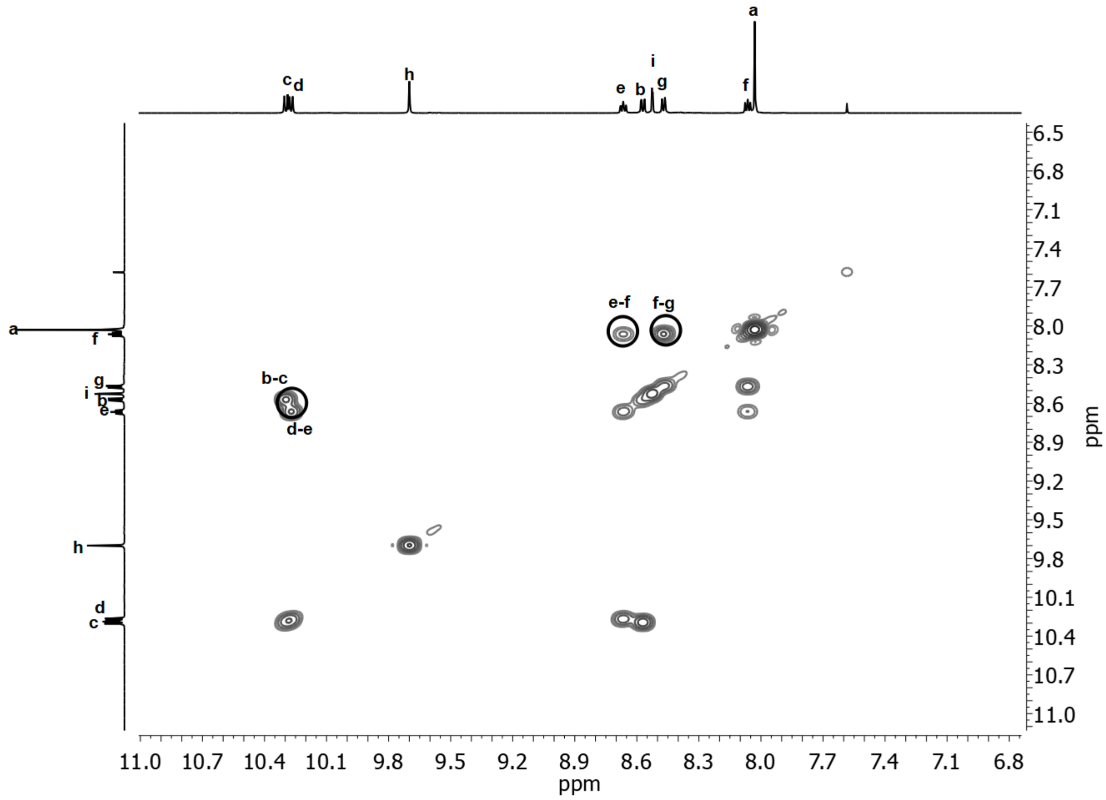

Figure S19 Partial $^1H - ^1H$ COSY spectrum (600 MHz, 298 K, CD_3CN) of $[Pd_2\mathbf{L^4}_2(MeCN)_4]^{4+}$.

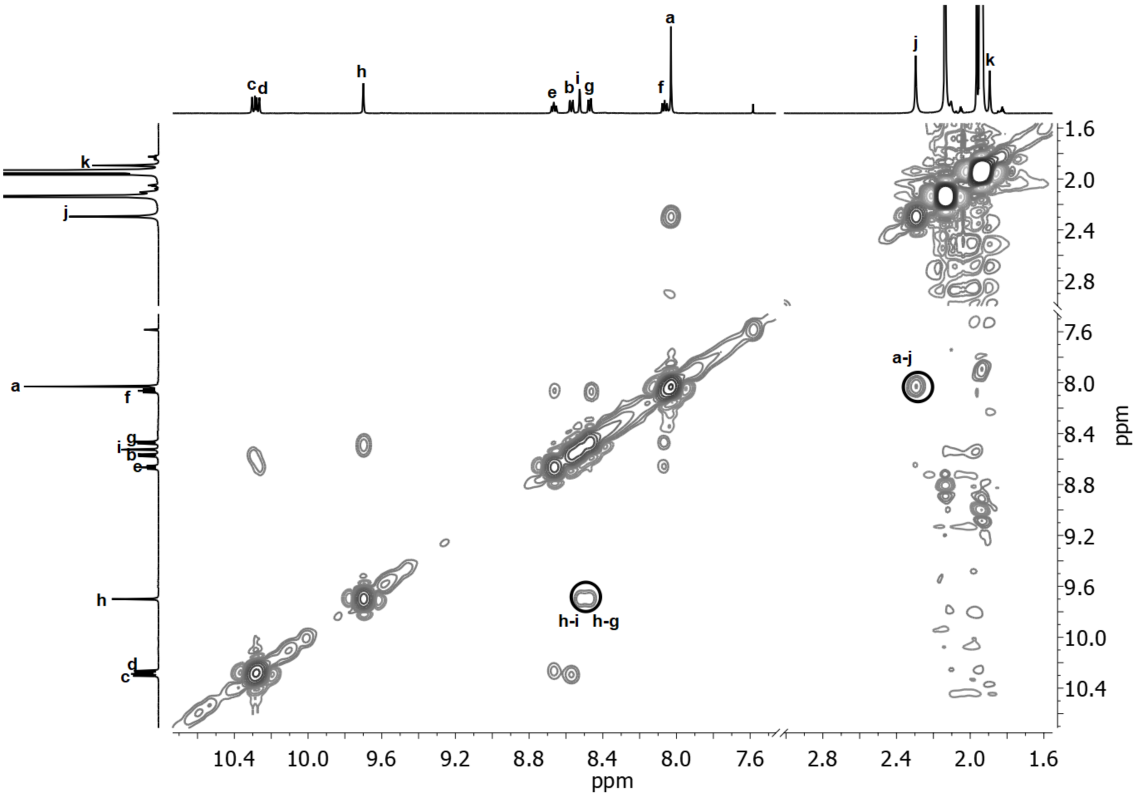

Figure S20 Partial $^1H - ^1H$ NOESY spectrum (600 MHz, 298 K, CD_3CN) of $[Pd_2\mathbf{L^4}_2(MeCN)_4]^{4+}$.

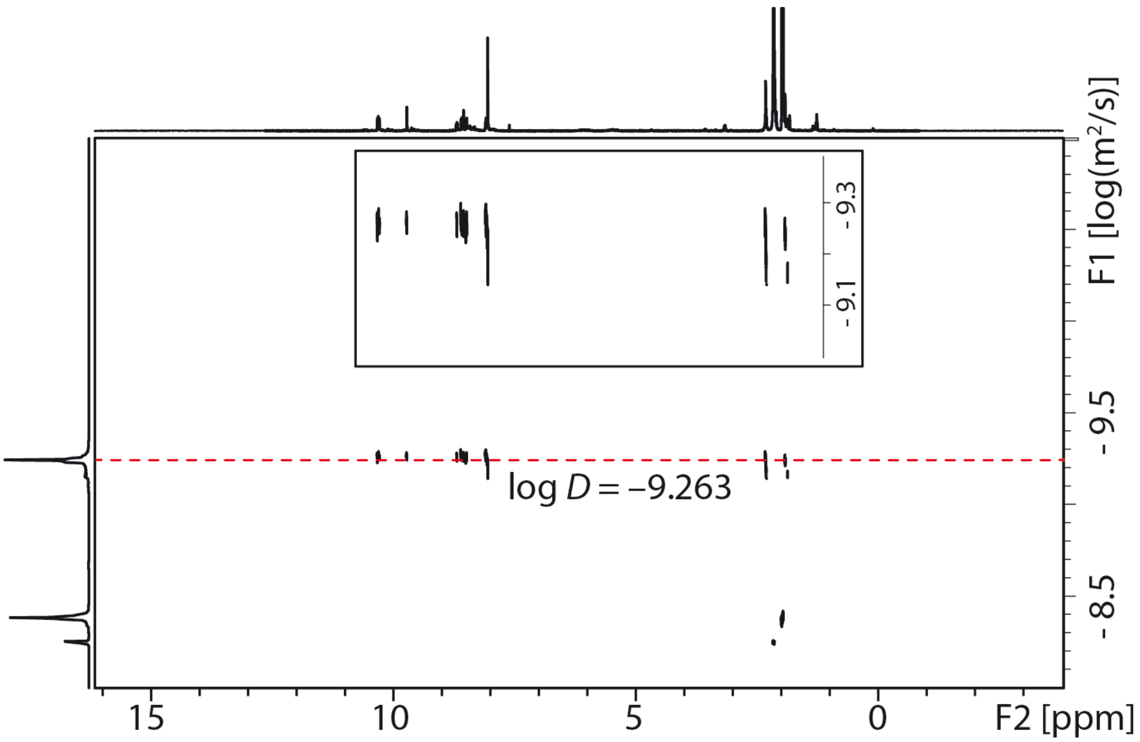

Figure S21 DOSY spectrum (500 MHz, 298 K, CD_3CN) of $[Pd_2\mathbf{L^4}_2(MeCN)_4]^{4+}$: diffusion coefficient = 5.5 x 10^{-10} m^2s^{-1}, log *D* = −9.26, r = 11.6 Å.

ESI HRMS ($C_{104}H_{72}N_{12}O_8Pd_2B_4F_{16}$): $[Pd_2\mathbf{L^4}_2(MeCN)_4]^{4+}$ calcd. for $C_{104}H_{72}N_{12}O_8Pd_2$ 457.5922; found 457.5932; $[Pd_2\mathbf{L^4}_2(MeCN)_3+F]^{3+}$ calcd. for $C_{102}H_{69}N_{11}O_8Pd_2F$ 602.7804; found 602.7814; $[Pd_2\mathbf{L^4}_2(MeCN)_2+2F]^{3+}$ calcd. for $C_{100}H_{66}N_{10}O_8Pd_2F_2$ 893.1568; found 893.1576.

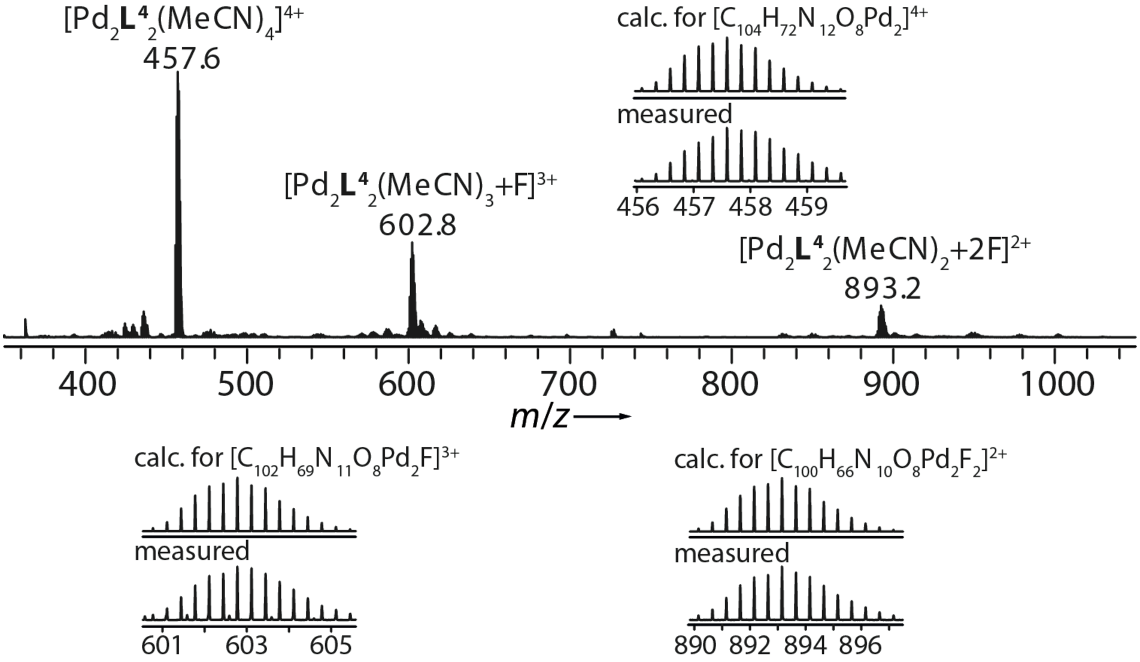

Figure S22 ESI mass spectrum of $[Pd_2\mathbf{L^4}_2(MeCN)_4]^{4+}$. The presence of the $[Pd_2\mathbf{L^4}_2(MeCN)_3+F]^{3+}$ and $[Pd_2\mathbf{L^4}_2(MeCN)_2+2F]^{2+}$ species is due to substitution of coordinated CH_3CN by traces of fluoride anions under the measurement conditions.

3.4 Titration of ring $[Pd_2L^4_2(MeCN)_4]^{4+}$ with chloride anions

A 600 µL solution of the ring $[Pd_2\mathbf{L^4}_2(MeCN)_4]^{4+}$ (0.64 mM) in CD_3CN was titrated with a concentrated solution of tetrabutylammonium chloride (NBu_4Cl) (30 mM) in CD_3CN. Upon each addition, the solution was shaken before acquiring the spectrum, which allowed equilibrium to be reached.

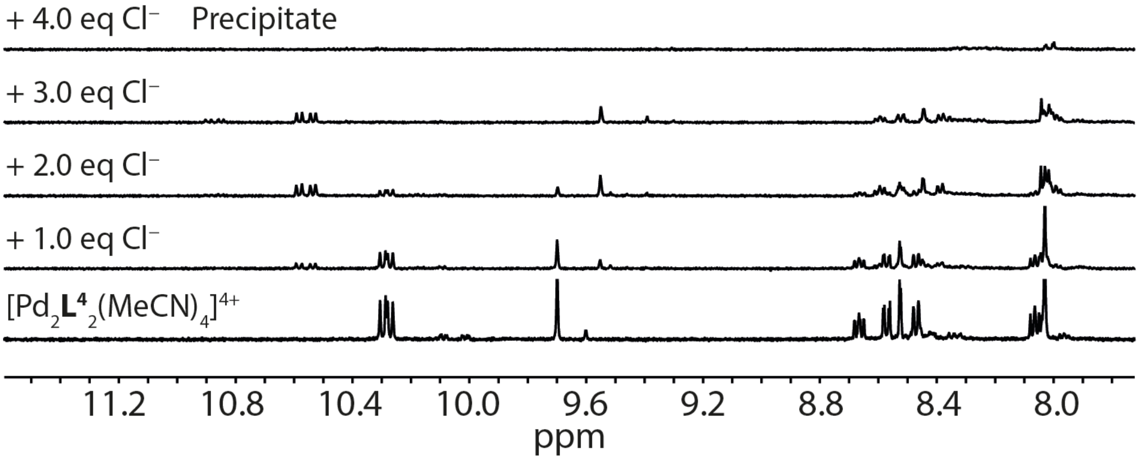

Figure S23 ^{1}H NMR titration (500 MHz, 298 K, CD_3CN) of $[Pd_2\mathbf{L^4}_2(MeCN)_4]^{4+}$ with NBu_4Cl. Upon addition of four equivalents of chloride, charged ring $[Pd_2\mathbf{L^4}_2(MeCN)_4]^{4+}$ transforms into neutral ring $[Pd_2\mathbf{L^4}_2Cl_4]$ which was found to precipitate from the polar solvent. The characterization of ring $[Pd_2\mathbf{L^4}_2Cl_4]$ is described below in detail.

3.5 Ring $[Pd_2L^4_2Cl_4]$

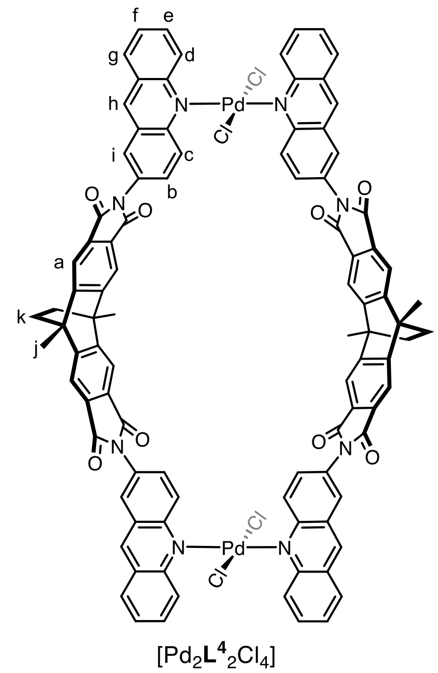

$[Pd_2\mathbf{L^4}_2Cl_4]$

A CD_3CN solution of the ring $[Pd_2\mathbf{L^4}_2(MeCN)_4]^{4+}$ (5080 µL, 0.64 mM, 3.25 µmol, 1 eq.) was mixed with CD_3CN solution of NBu_4Cl (433 µL, 30 mM, 13.00 µmol, 4 eq.) at room temperature for several minutes to give the neutral compound $[Pd_2\mathbf{L^4}_2Cl_4]$ as precipitate. The product was collected via centrifugation, washed with pure chloroform and dried in vacuum

to give a yellow solid (4.0 mg, 68%). The solid is soluble in DMSO and DMF, however, proton signals of the free ligand and a second species (presumably mono-coordinated ligand) were found to arise after standing for several hours.

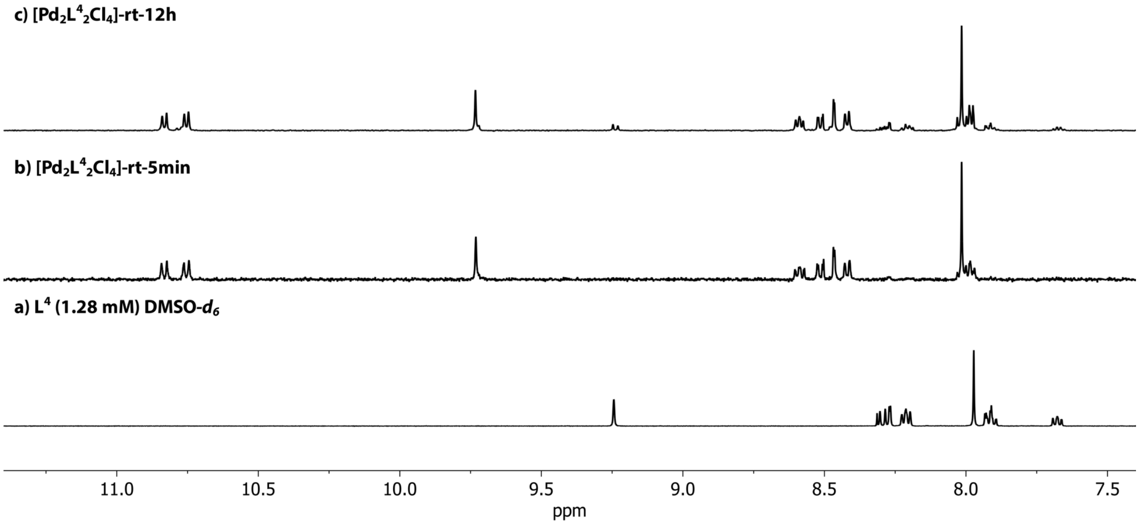

Figure S24 ^{1}H NMR spectra (298 K, DMSO-d_6) of (a) **L**^{4} (1.28mM), (b) and (c) re-dissolved [Pd_2**L**$^4_2Cl_4$] in DMSO-d_6 for 5min or 12 h at room temperature, indicating decomposition of [Pd_2**L**$^4_2Cl_4$] in DMSO.

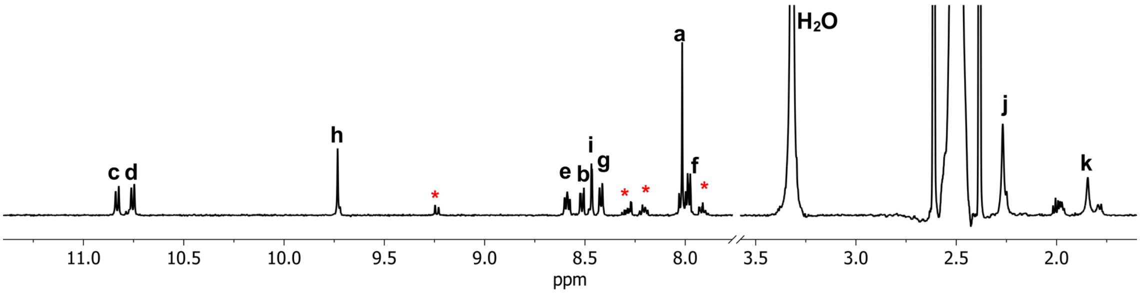

Figure S25 ^{1}H NMR spectrum (600 MHz, 298 K, DMSO-d_6) of [Pd_2**L**$^4_2Cl_4$]. Asterisks represent proton signals of released ligand after standing for several hours during the 2D NMR experiments.

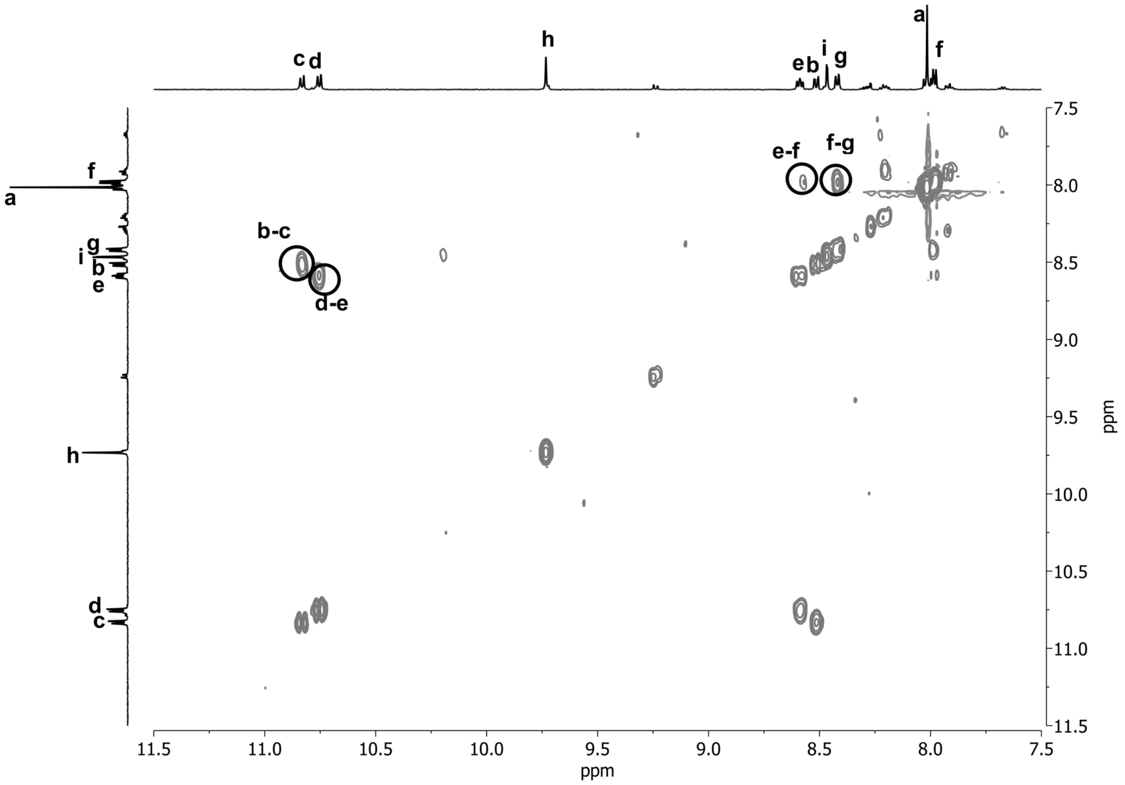

Figure S26 Partial ^{1}H – ^{1}H COSY spectrum (600 MHz, 298 K, DMSO-d_6) of [$Pd_2\mathbf{L^4}_2Cl_4$].

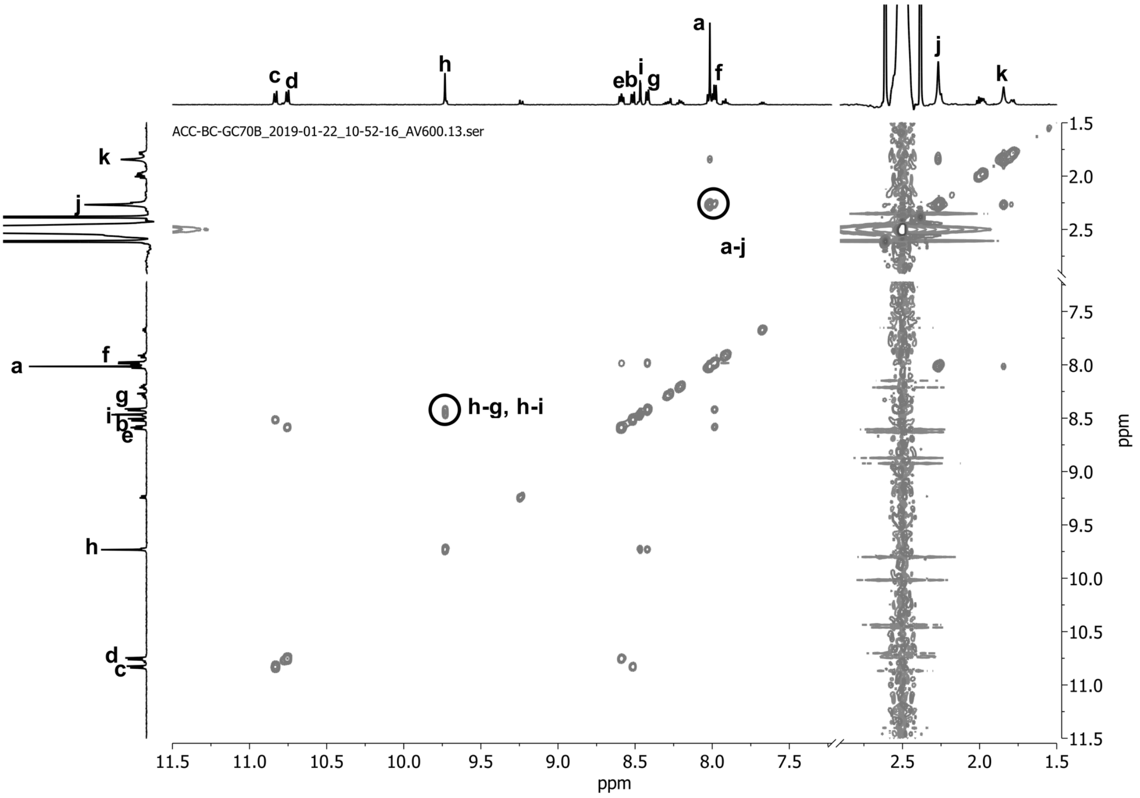

Figure S27 Partial ^{1}H – ^{1}H NOESY spectrum (600 MHz, 298 K, DMSO-d_6) of [$Pd_2\mathbf{L^4}_2Cl_4$].

4 Fullerene binding studies

General procedure: To a CD_3CN solution of the host compounds (0.64 mM for $[Pd_2\mathbf{L}^3{}_4]^{4+}$ and $[Pd_2\mathbf{L}^4{}_2(MeCN)_4]^{4+}$) in a sealed vessel, excess fullerene (C_{60}, C_{70}) was added as finely grounded powders. The mixtures were sonicated for 3 minutes, then stirred at room temperature or left standing at 70 °C for several days. Upon cooling, the supernatant was collected and transferred to NMR tubes.

4.1 Fullerene binding experiment with cage $[Pd_2L^3{}_4]^{4+}$

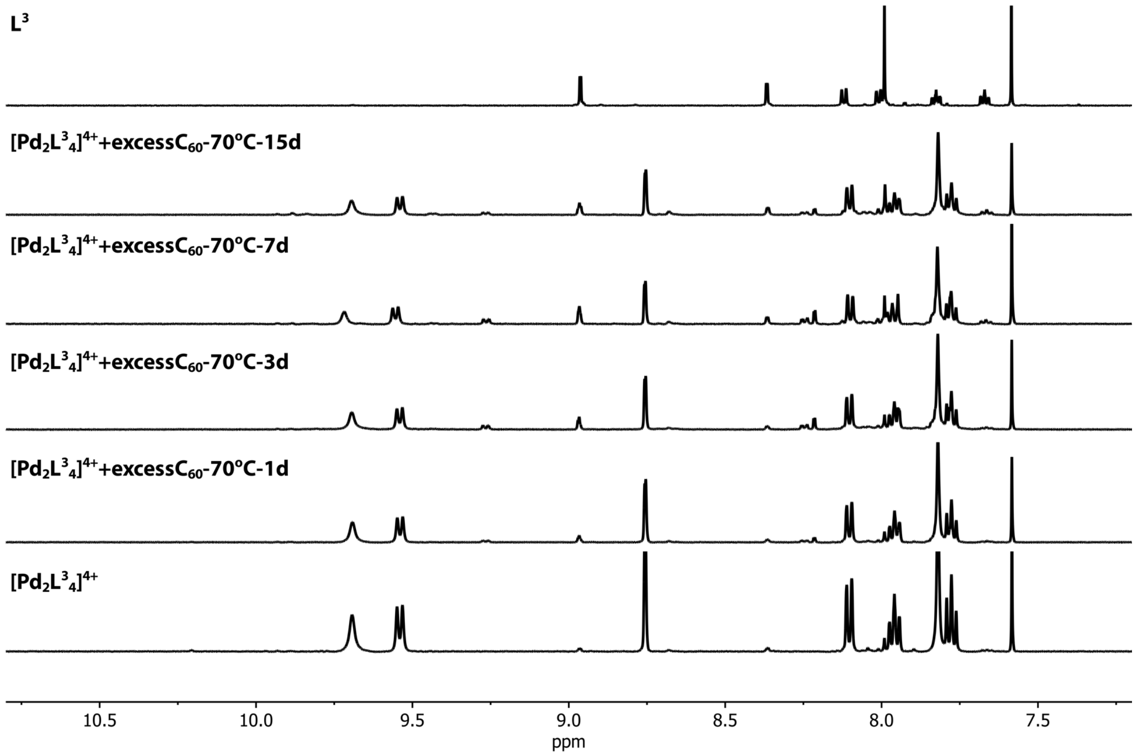

Figure S28 ^1H NMR spectra (500 MHz, 298 K, CD_3CN) monitoring the test of binding C_{60} in $[Pd_2\mathbf{L}^3{}_4]^{4+}$ at 70 °C, indicating that C_{60} cannot be encapsulated in $[Pd_2\mathbf{L}^3{}_4]^{4+}$ and partial decomposition of $[Pd_2\mathbf{L}^3{}_4]^{4+}$.

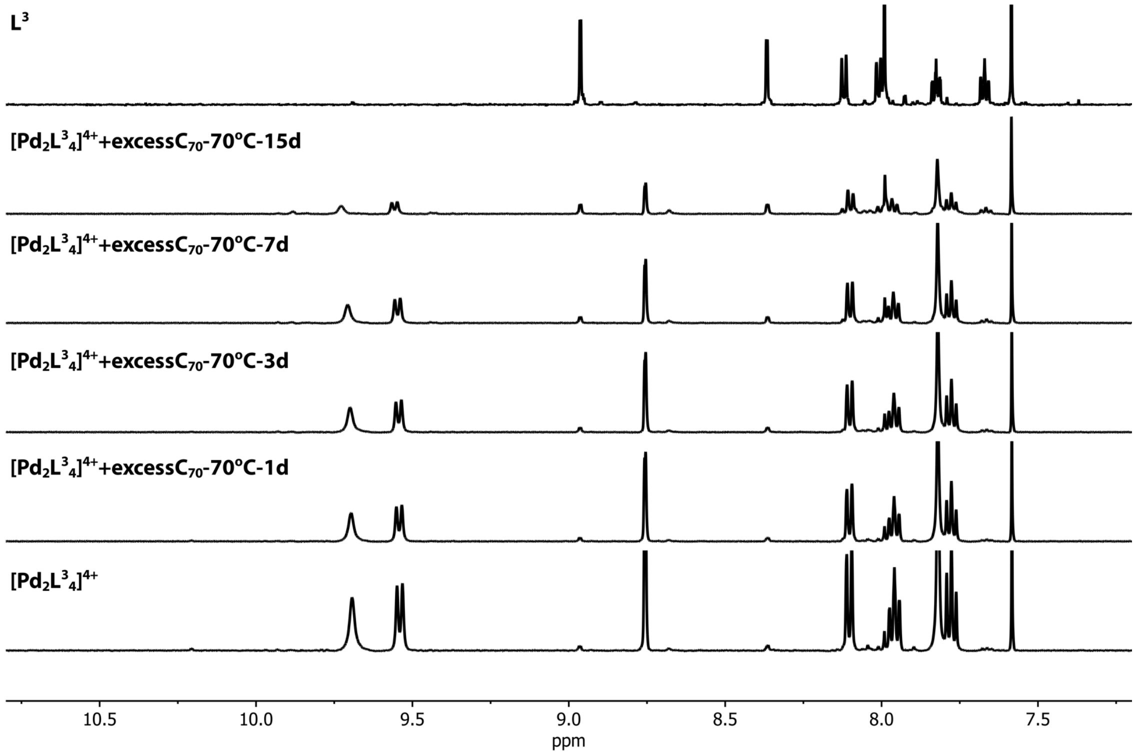

Figure S29 ^{1}H NMR spectra (500 MHz, 298 K, CD_3CN) monitoring the test of binding C_{70} in $[Pd_2\mathbf{L^3}_4]^{4+}$ at 70 ^{o}C, indicating that C_{70} cannot be encapsulated in $[Pd_2\mathbf{L^3}_4]^{4+}$ and partial decomposition of $[Pd_2\mathbf{L^3}_4]^{4+}$.

4.2 Fullerene binding experiment with ring $[Pd_2L^4_2(MeCN)_4]^{4+}$

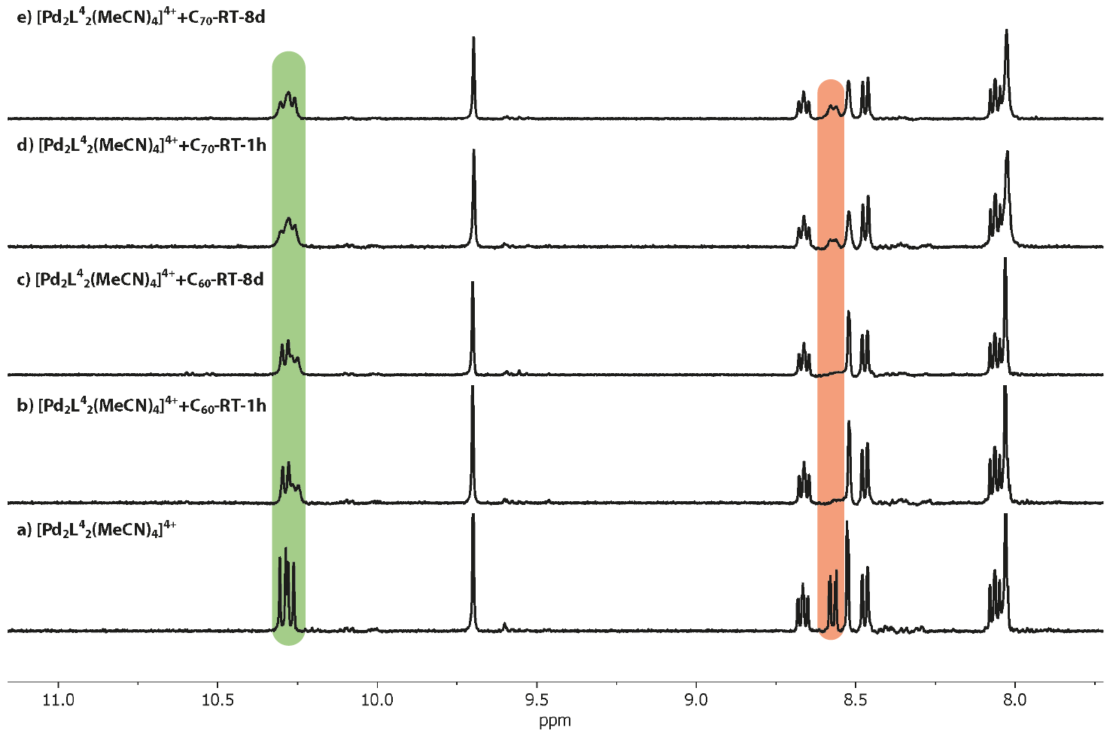

Figure S30 ^{1}H NMR spectra (500 MHz, 298 K, CD_3CN) monitoring the test of binding C_{60}/C_{70} in $[Pd_2\mathbf{L^4}_2(MeCN)_4]^{4+}$ at room temperature for 1 h or 8 d, indicating fast exchange between ring and C_{60}/C_{70}. The acridine protons (H_c, H_d) of ring $[Pd_2\mathbf{L^4}_2(MeCN)_4]^{4+}$ and proton H_b are highlighted in green and red, respectively.

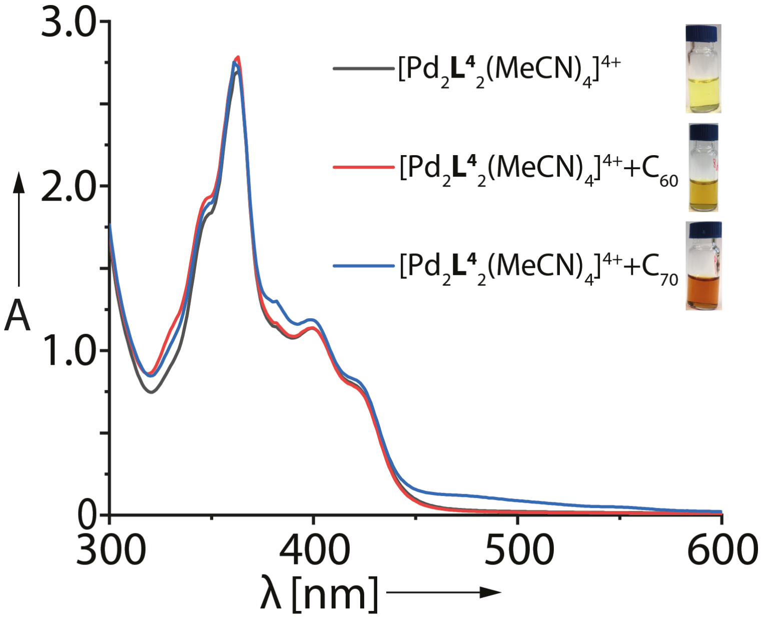

Figure S31 UV-Vis spectra (0.64 mM, CH_3CN, 298 K) and photographs of $[Pd_2\mathbf{L^4}_2(MeCN)_4]^{4+}$ in CH_3CN with/without fullerenes.

5 X-Ray data

5.1 General methods

Four different supramolecular assemblies [C_{70}@Pd_2**L^2**$_4$](BF_4)$_4$, [Pd_2**L^3**$_4$](BF_4)$_4$, [Pd_2**L^4**$_2Cl_4$] and [Pd_2**L^4**$_2Cl_4$]_B (another crystalline form of ring [Pd_2**L^4**$_2Cl_4$]) were studied using single crystal X-ray crystallography. The crystals of all supramolecular assemblies were extremely sensitive to loss of organic solvent. Due to the very thin (5 – 20 µm) plate-like crystals, the analysis was hampered by the limited scattering power of the samples not allowing to reach the desired (sub-)atomic resolution using a modern microfocussed in-house X-ray CuK_α source. Gaining detailed structural insight thus required cryogenic crystal handling and highly brilliant synchrotron radiation. Hence, diffraction data of all supramolecular assemblies was collected during three beamtime shifts at macromolecular synchrotron beamline P11, PETRA III, DESY.[3] Modelling of C_{70} disorder as well as counterion and solvent flexibility required carefully adapted macromolecular refinement protocols employing geometrical restraint dictionaries, similarity restraints and restraints for anisotropic displacement parameters (ADPs).

Table S1 Crystallographic data of [C_{70}@Pd_2**L^2**$_4$](BF_4)$_4$ and [Pd_2**L^3**$_4$](BF_4)$_4$.

Compound	**[C_{70}@$Pd_2L^2_4$](BF_4)$_4$**	**[$Pd_2L^3_4$](BF_4)$_4$**
CCDC number	1939201	1939202
Identification code	bc19d_sav_sq	bc4a_sq
Empirical formula	$C_{246}H_{136}N_{16}O_{24}Pd_2B_4F_{16}$	$C_{168}H_{116}N_{20}O_{16}Pd_2B_3F_{12}$
Formula weight	4259.74	3144.03
Temperature (K)	80(2)	80(2)
Crystal system	Monoclinic	Tetragonal
Space group	$P2_1/n$	$P4/ncc$
a (Å)	16.617(3)	18.445(3)
b (Å)	19.654(4)	18.445(3)
c (Å)	31.710(6)	51.044(10)
α (º)	90	90
β (º)	101.53(3)	90
γ (º)	90	90
Volume (Å^3)	10147(4)	17366(6)
Z	2	4
Density (calc.) (g/cm^3)	1.394	1.203
Absorption coefficient (mm^{-1})	0.244	0.258
F(000)	4344	6428
Crystal size (mm^3)	0.100 x 0.040 x 0.020	0.100 x 0.080 x 0.040
θ range for data collection (º)	1.188 to 22.490	0.773 to 20.563
Reflections collected	98808	113076

Observed reflections [R(int)]	14491 [0.0377]	4820 [0.1434]
Goodness-of-fit on F^2	1.638	1.052
R_1 [I>2σ(I)]	0.1174	0.1306
wR_2 (all data)	0.3936	0.3537
Largest diff. peak and hole (e.Å^{-3})	0.920 and -0.788	1.382 and -1.000
Data / restraints / parameters	14491 / 10841 / 1752	4820 / 1398 / 601

Table S2 Crystallographic data of [Pd_2**L^4**$_2Cl_4$] and [Pd_2**L^4**$_2Cl_4$]_B.

Compound	**[$Pd_2L^2{}_2Cl_4$]**	**[$Pd_2L^2{}_2Cl_4$]_B**
CCDC number	1939203	1939204
Identification code	bc23a_sq	bc23b_sq
Empirical formula	$C_{96}H_{60}N_8O_8Pd_2Cl_4$	$C_{96}H_{60}N_8O_8Pd_2Cl_4$
Formula weight	1808.12	1808.12
Temperature (K)	80(2)	80(2)
Crystal system	Triclinic	Monoclinic
Space group	*P*-1	*P*2$_1$/c
a (Å)	12.008(2)	22.976(5)
b (Å)	22.155(4)	36.611(7)
c (Å)	24.669(5)	14.926(3)
α (º)	64.69(3)	90
β (º)	84.73(3)	104.03(3)
γ (º)	78.34(3)	90
Volume (Å^3)	5810(2)	12181(4)
Z	2	4
Density (calc.) (g/cm^3)	1.033	0.986
Absorption coefficient (mm^{-1})	0.406	0.387
F(000)	1832	3664
Crystal size (mm^3)	0.080x 0.040 x 0.020	0.090 x 0.040 x 0.005
θ range for data collection (º)	0.885 to 15.359	0.885 to 20.144
Reflections collected	18212	46112
Observed reflections [R(int)]	5322 [0.1197]	12709 [0.0866]
Goodness-of-fit on F^2	1.632	1.173
R_1 [I>2σ(I)]	0.1607	0.1044
wR_2 (all data)	0.4359	0.3630
Largest diff. peak and hole (e.Å^{-3})	1.617 and -0.576	1.437 and -0.746
Data / restraints / parameters	5322 / 2719 / 1067	12709 / 2360 / 1067

5.2 Crystal structure of [C_{70}@Pd_2**L**$^2_4$](BF_4)$_4$

Red plate-shaped crystals of [C_{70}@Pd_2**L**$^2_4$](BF_4)$_4$ were obtained by slow vapor diffusion of ethyl acetate into a 0.64 mM CD_3CN solution of [C_{70}@Pd_2**L**$^2_3$(MeCN)$_2$](BF_4)$_4$. A single crystal in mother liquor was pipetted onto a glass slide containing NVH oil. To avoid collapse of the crystal lattice, the crystal was quickly mounted onto a 0.1 mm nylon loop and immediately flash cooled in liquid nitrogen. Crystals were stored at cryogenic temperature in dry shippers, in which they were safely transported to macromolecular beamline P11 at Petra III[3], DESY, Germany.

A wavelength of λ = 0.6888 Å was chosen using a liquid N_2 cooled double crystal monochromator. Single crystal X-ray diffraction data was collected at 80(2) K on a single axis goniometer, equipped with an Oxford Cryostream 800 and a Pilatus 6M detector. 1800 diffraction images were collected in a 360° ϕ sweep at a detector distance of 156 mm, 30% filter transmission, 0.2° step width and 0.2 seconds exposure time per image. Data integration and reduction were undertaken using XDS.[4] The structure was solved by intrinsic phasing/direct methods using SHELXT[5] and refined with SHELXL[6] using 22 cpu cores for full-matrix least-squares routines on F^2 and ShelXle[7] as a graphical user interface and the DSR program plugin was employed for modeling.[8]

5.2.1 Specific refinement details of [C_{70}@Pd_2L$^2_4$](BF_4)$_4$

A C_2 symmetry element is located at the center of the complex. Stereochemical restraints for the EAQ ligands (**L^2**) and ethyl acetate (OAC) were generated by the GRADE program using the GRADE Web Server (http://grade.globalphasing.org) and applied in the refinement. A GRADE dictionary for SHELXL contains target values and standard deviations for 1,2-distances (DFIX) and 1,3-distances (DANG), as well as restraints for planar groups (FLAT). All displacements for non-hydrogen atoms were refined anisotropically. The refinement of ADP's for carbon, nitrogen and oxygen atoms was enabled by a combination of similarity restraints (SIMU) and rigid bond restraints (RIGU).[9] Disorder of C_{70} guest was modelled with two discrete positions each using the DSR program GUI and its SADI restraints for 1,2-distances, 1,3-distances and planar groups for C_{70}.[8, 10] The contribution of the electron density from disordered counterions and solvent molecules, which could not be modeled with discrete atomic positions, were handled using the SQUEEZE[11] routine in PLATON.[12] The solvent mask file (.fab) computed by PLATON were included in the SHELXL refinement via the ABIN instruction leaving the measured intensities untouched.

Table S3 Definition of residues involved in [C_{70}@Pd_2**L**$^2_4$](BF_4)$_4$.

Fragment	Residue class	Occurrence	Residue numbers
Pd^{2+}		1	1
Ligand **L^2**	EAQ	2	2,3
C_{70}	C70	1	4 (50% occupation)
BF_4^-	BF4	2	5,6 (disordered)
Ethyl acetate	OAC	2	8,9

5.2.2 Description of the structure of [C_{70}@$Pd_2L^2_4$](BF_4)$_4$

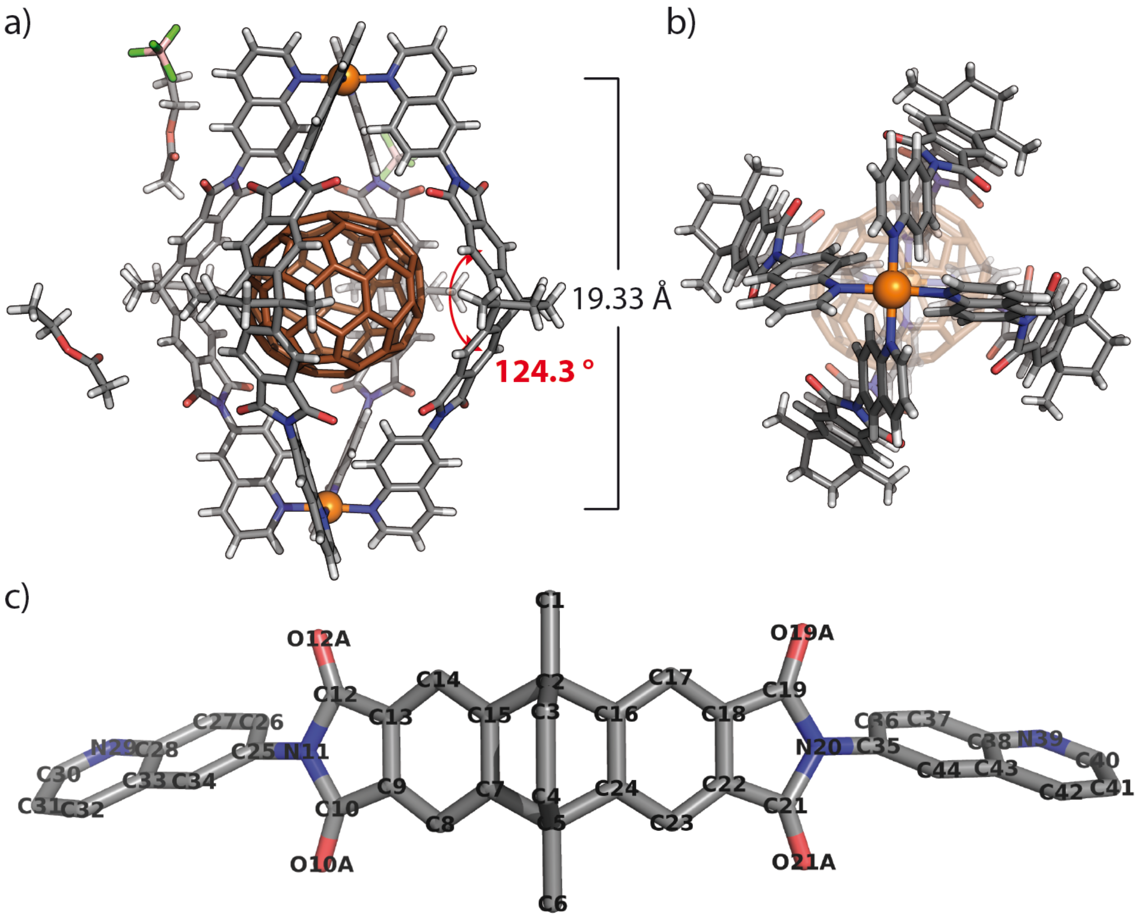

Figure S32 X-ray structure of [C_{70}@Pd_2**L**$^2_4$](BF_4)$_4$: (a) full structure showing the Pd–Pd distance of 19.33 Å; (b) top view; (c) atom naming scheme of ligand **L**^{2} (residue class EAQ). The same atom labels are used in all other **L**^{2} containing structures. Color scheme: H, light grey; B, pink; C, dark grey; N, blue; O, red; F, green; Pd, orange; C_{70}: brown.

Table S4 Structural details involved in [C_{70}@Pd_2**L**$^2_4$](BF_4)$_4$.

Residues No.	Dihedral angle (°) between the backbone's benzene planes C16_C17_C18_C22_C23_C24 and C7_C8_C9_C13_C14_C15	Esd (°)	Dihedral angle (°) between planes N29_Pd1_Pd2 and N39_Pd1_Pd2	Esd (°)
2	55.185	0.230	0.685	0.275
3	56.143	0.321	2.065	0.358
Average	55.7		1.4	

5.3 Crystal structure of $[Pd_2L^3_4](BF_4)_4$

Colorless plate-shaped crystals of $[Pd_2\mathbf{L^3}_4](BF_4)_4$ were obtained by slow vapor diffusion of methyl tert-butyl ether into a 0.64 mM CD_3CN solution of $[Pd_2\mathbf{L^3}_4](BF_4)_4$. A single crystal in mother liquor was pipetted onto a glass slide containing NVH oil. To avoid collapse of the crystal lattice, the crystal was quickly mounted onto a 0.1 mm nylon loop and immediately flash cooled in liquid nitrogen. Crystals were stored at cryogenic temperature in dry shippers, in which they were safely transported to macromolecular beamline P11 at Petra III[3], DESY, Germany.

A wavelength of λ = 0.6888 Å was chosen using a liquid N_2 cooled double crystal monochromator. Single crystal X-ray diffraction data was collected at 80(2) K on a single axis goniometer, equipped with an Oxford Cryostream 800 and a Pilatus 6M detector. 1800 diffraction images were collected in a 360° ϕ sweep at a detector distance of 156 mm, 30% filter transmission, 0.2° step width and 0.2 seconds exposure time per image. Data integration and reduction were undertaken using XDS.[4] The structure was solved by intrinsic phasing/direct methods using SHELXT[5] and refined with SHELXL[6] using 22 cpu cores for full-matrix least-squares routines on F^2 and ShelXle[7] as a graphical user interface and the DSR program plugin was employed for modeling.[8]

5.3.1 Specific refinement details of $[Pd_2L^3_4](BF_4)_4$

The occupancies of two Pd atoms and three BF_4 moieties were refined with 0.25 owing to the C_4 symmetry element oriented along the Pd–Pd axis. Stereochemical restraints for the ETQ ligands (**L³**) were generated by the GRADE program using the GRADE Web Server (http://grade.globalphasing.org) and applied in the refinement. A GRADE dictionary for SHELXL contains target values and standard deviations for 1,2-distances (DFIX) and 1,3-distances (DANG), as well as restraints for planar groups (FLAT). All displacements for non-hydrogen atoms were refined anisotropically. The refinement of ADP's for carbon, nitrogen and oxygen atoms was enabled by a combination of similarity restraints (SIMU) and rigid bond restraints (RIGU).[9] The contribution of the electron density from disordered counterions and solvent molecules, which could not be modeled with discrete atomic positions, were handled using the SQUEEZE[11] routine in PLATON.[12] The solvent mask file (.fab) computed by PLATON were included in the SHELXL refinement via the ABIN instruction leaving the measured intensities untouched.

Table S5 Definition of residues involved in $[Pd_2\mathbf{L^3}_4](BF_4)_4$.

Fragment	Residue class	Occurrence	Residue numbers
Pd^{2+}	PD	1	1 (25 % occupation)
Ligand **L³**	ETQ	1	2 (25 % occupation)
BF_4^-	BF4	3	3, 4, 5 (25 % occupation)
Acetonitrile	ACN	1	6

5.3.2 Description of the structure of $[Pd_2L^3{}_4](BF_4)_4$

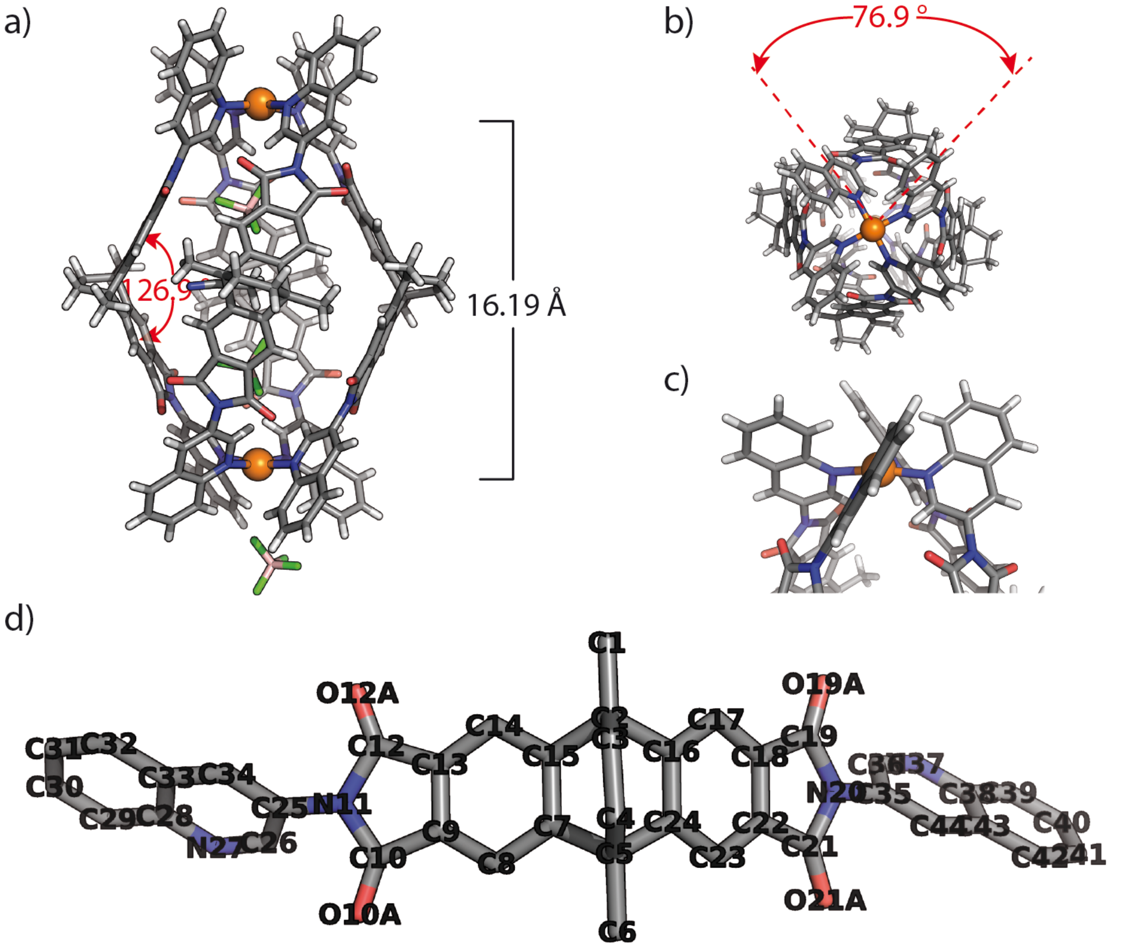

Figure S33 X-ray structure of $[Pd_2\mathbf{L^3}_4](BF_4)_4$: (a) full structure showing the Pd–Pd distance of 16.19 Å; (b) top view depicting a dihedral angle of 76.9° in between two pyridine arms of the same ligand; (c) coordination center showing a highly twisted geometry due to steric hindrance from hydrogen atoms of quinoline moieties; (d) atom naming scheme of ligand $\mathbf{L^3}$ (residue class ETQ). Color scheme: H, light grey; B, pink; C, dark grey; N, blue; O, red; F, green; Pd, orange.

Table S6 Structural details involved in $[Pd_2\mathbf{L^3}_4](BF_4)_4$.

Residues No.	Dihedral angle (°) between the backbone's benzene planes C16_C17_C18_C22_C23_C24 and C7_C8_C9_C13_C14_C15	Esd (°)	Dihedral angle (°) between planes N27_Pd1_Pd2 and N37_Pd1_Pd2	Esd (°)
2	53.130	0.432	76.929	0.322

5.4 Crystal structure of $[Pd_2L^4{}_2Cl_4]$

Colorless plate-shaped crystals of $[Pd_2\mathbf{L^4}_2Cl_4]$ were obtained by slow vapor diffusion of benzene into a 0.64 mM CD_3CN solution (100 µL) of $[Pd_2\mathbf{L^4}_2(MeCN)_4](BF_4)_4$ in the presence of 5 eq. of tetrabutylammonium periodate ($TBAIO_4$). A single crystal in mother liquor was pipetted onto a glass slide containing NVH oil. To avoid collapse of the crystal lattice, the

crystal was quickly mounted onto a 0.1 mm nylon loop and immediately flash cooled in liquid nitrogen. Crystals were stored at cryogenic temperature in dry shippers, in which they were safely transported to macromolecular beamline P11 at Petra III[3], DESY, Germany.

A wavelength of λ = 0.6888 Å was chosen using a liquid N_2 cooled double crystal monochromator. Single crystal X-ray diffraction data was collected at 80(2) K on a single axis goniometer, equipped with an Oxford Cryostream 800 and a Pilatus 6M detector. 1800 diffraction images were collected in a 360° ϕ sweep at a detector distance of 156 mm, 30% filter transmission, 0.2° step width and 0.2 seconds exposure time per image. Data integration and reduction were undertaken using XDS.[4] The structure was solved by intrinsic phasing/direct methods using SHELXT[5] and refined with SHELXL[6] using 22 cpu cores for full-matrix least-squares routines on F^2 and ShelXle[7] as a graphical user interface and the DSR program plugin was employed for modeling.[8]

5.4.1 Specific refinement details of [Pd_2**L**$^4{}_2$$Cl_4$]

The chloride atoms were assigned crystallographically by the electron density and the bond distances (Pd–Cl: 2.33 Å), although chloride anions were not meant to be contained in the solution of sample [Pd_2**L**$^4{}_2$(MeCN)$_4$](BF_4)$_4$. We presume that the observed chloride ions came from a contamination in the added $TBAIO_4$ or the partial decomposition of $CHCl_3$ which was used in the last purification step for ligand **L**^{4}. Stereochemical restraints for the EAA ligands (**L**^{4}) were generated by the GRADE program using the GRADE Web Server (http://grade.globalphasing.org) and applied in the refinement. A GRADE dictionary for SHELXL contains target values and standard deviations for 1,2-distances (DFIX) and 1,3-distances (DANG), as well as restraints for planar groups (FLAT). All displacements for non-hydrogen atoms were refined anisotropically. The refinement of ADP's for carbon, nitrogen and oxygen atoms was enabled by a combination of similarity restraints (SIMU) and rigid bond restraints (RIGU).[9] The contribution of the electron density from disordered counterions and solvent molecules, which could not be modeled with discrete atomic positions, were handled using the SQUEEZE[11] routine in PLATON.[12] The solvent mask file (.fab) computed by PLATON were included in the SHELXL refinement via the ABIN instruction leaving the measured intensities untouched.

Table S7 Definition of residues involved in [Pd_2**L**$^4{}_2$$Cl_4$].

Fragment	Residue class	Occurrence	Residue numbers
Pd^{2+}	PD	1	1
Ligand **L**^{4}	EAA	2	2, 3
Cl^-		1	4

5.4.2 Description of the structure of [$Pd_2L^4{}_2Cl_4$]

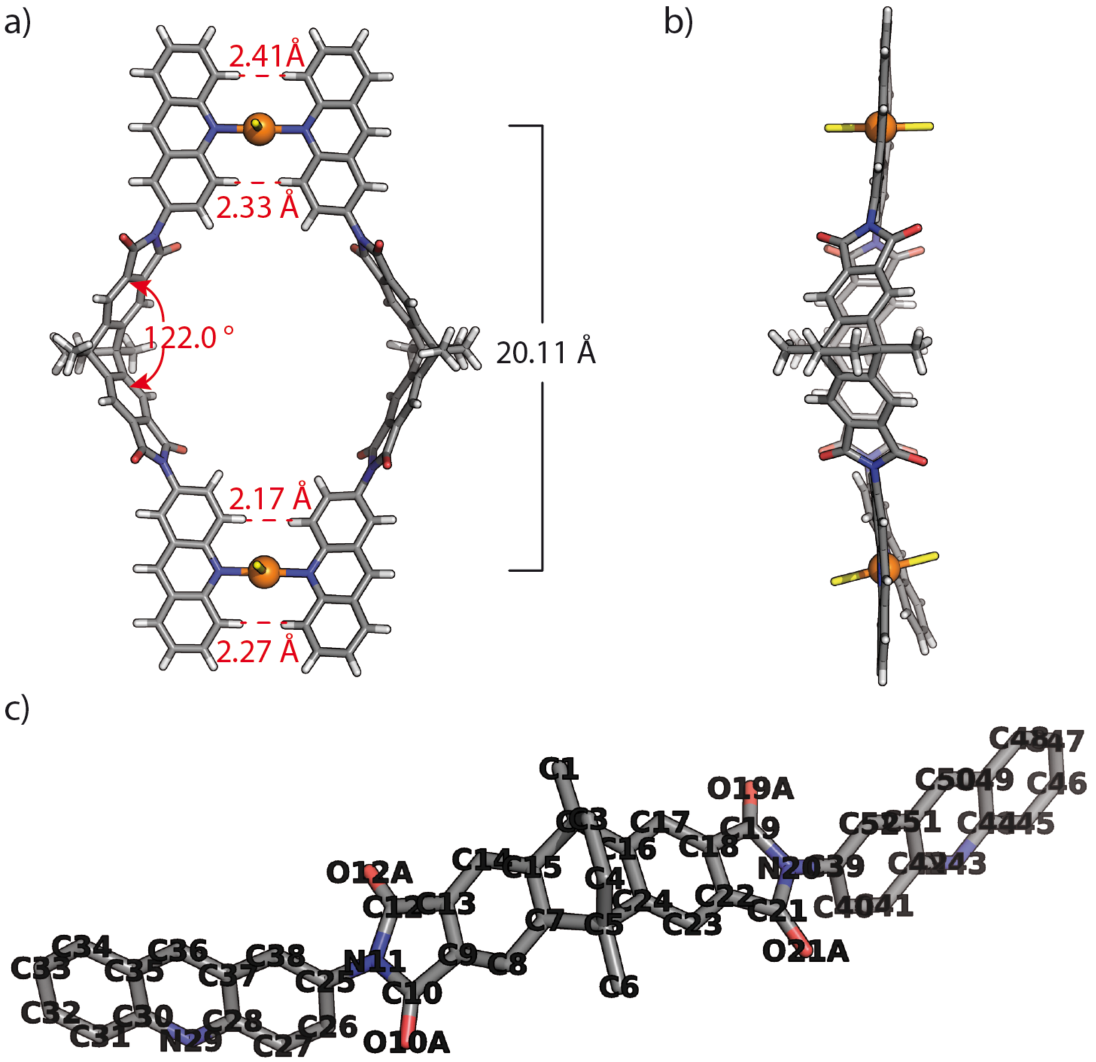

Figure S34 X-ray structure of [Pd_2**L**$^4{}_2Cl_4$]: (a) full structure showing the Pd–Pd distance of 20.11 Å; (b) equatorial view; (c) atom naming scheme of ligand **L**^{4} (residue class EAA). Color scheme: H, light grey; C, dark grey; N, blue; O, red; Cl, yellow; Pd, orange.

Table S8 Structural details involved in [Pd_2**L**$^4{}_2Cl_4$].

Residues No.	Dihedral angle (°) between the backbone's benzene planes C16_C17_C18_C22_C23_C24 and C7_C8_C9_C13_C14_C15	Esd (°)	Dihedral angle (°) between planes N29_Pd1_Pd2 and N43_Pd1_Pd2	Esd (°)
2	57.732	0.933	3.850	0.337
3	58.288	0.805	4.279	0.346
Average	58.0		4.1	

5.5 Crystal structure of [Pd_2**L**$^4{}_2$$Cl_4$]_B

Colorless plate-shaped crystals of [Pd_2**L**$^4{}_2$$Cl_4$]_B were obtained by slow vapor diffusion of benzene into a 0.64 mM CD_3CN solution (100 µL) of [Pd_2**L**$^4{}_2$$(MeCN)_4$]$(BF_4)_4$ in the presence of 10 eq. of tetrabutylammonium periodate ($TBAIO_4$). A single crystal in mother liquor was pipetted onto a glass slide containing NVH oil. To avoid collapse of the crystal lattice, the crystal was quickly mounted onto a 0.1 mm nylon loop and immediately flash cooled in liquid nitrogen. Crystals were stored at cryogenic temperature in dry shippers, in which they were safely transported to macromolecular beamline P11 at Petra III[3], DESY, Germany.

A wavelength of λ = 0.6888 Å was chosen using a liquid N_2 cooled double crystal monochromator. Single crystal X-ray diffraction data was collected at 80(2) K on a single axis goniometer, equipped with an Oxford Cryostream 800 and a Pilatus 6M detector. 1900 diffraction images were collected in a 360° ϕ sweep at a detector distance of 200 mm, 100% filter transmission, 0.2° step width and 0.1 seconds exposure time per image. Data integration and reduction were undertaken using XDS.[4] The structure was solved by intrinsic phasing/direct methods using SHELXT[5] and refined with SHELXL[6] using 22 cpu cores for full-matrix least-squares routines on F^2 and ShelXle[7] as a graphical user interface and the DSR program plugin was employed for modeling.[8]

5.5.1 Specific refinement details of [Pd_2**L**$^4{}_2$$Cl_4$]_B

The chloride atoms were assigned crystallographically by the electron density and the bond distances (Pd–Cl: 2.33 Å), although chloride anions were not meant to be contained in the solution of sample [Pd_2**L**$^4{}_2$$(MeCN)_4$]$(BF_4)_4$. We presume that the observed chloride ions came from a contamination in the added $TBAIO_4$ or the partial decomposition of $CHCl_3$ which was used in the last purification step for ligand **L**^{4}. Stereochemical restraints for the EAA ligands (**L**^{4}) were generated by the GRADE program using the GRADE Web Server (http://grade.globalphasing.org) and applied in the refinement. A GRADE dictionary for SHELXL contains target values and standard deviations for 1,2-distances (DFIX) and 1,3-distances (DANG), as well as restraints for planar groups (FLAT). All displacements for non-hydrogen atoms were refined anisotropically. The refinement of ADP's for carbon, nitrogen and oxygen atoms was enabled by a combination of similarity restraints (SIMU) and rigid bond restraints (RIGU).[9] The contribution of the electron density from disordered counterions and solvent molecules, which could not be modeled with discrete atomic positions, were handled using the SQUEEZE[11] routine in PLATON.[12] The solvent mask file (.fab) computed by PLATON were included in the SHELXL refinement via the ABIN instruction leaving the measured intensities untouched.

Table S9 Definition of residues involved in [Pd_2**L**$^4{}_2$$Cl_4$]_B.

Fragment	Residue class	Occurrence	Residue numbers
Pd^{2+}	PD	1	1
Ligand **L**^{4}	EAA	2	2, 3
Cl^-	CL	1	4

5.5.2 Description of the structure of [$Pd_2L^4{}_2Cl_4$]_B

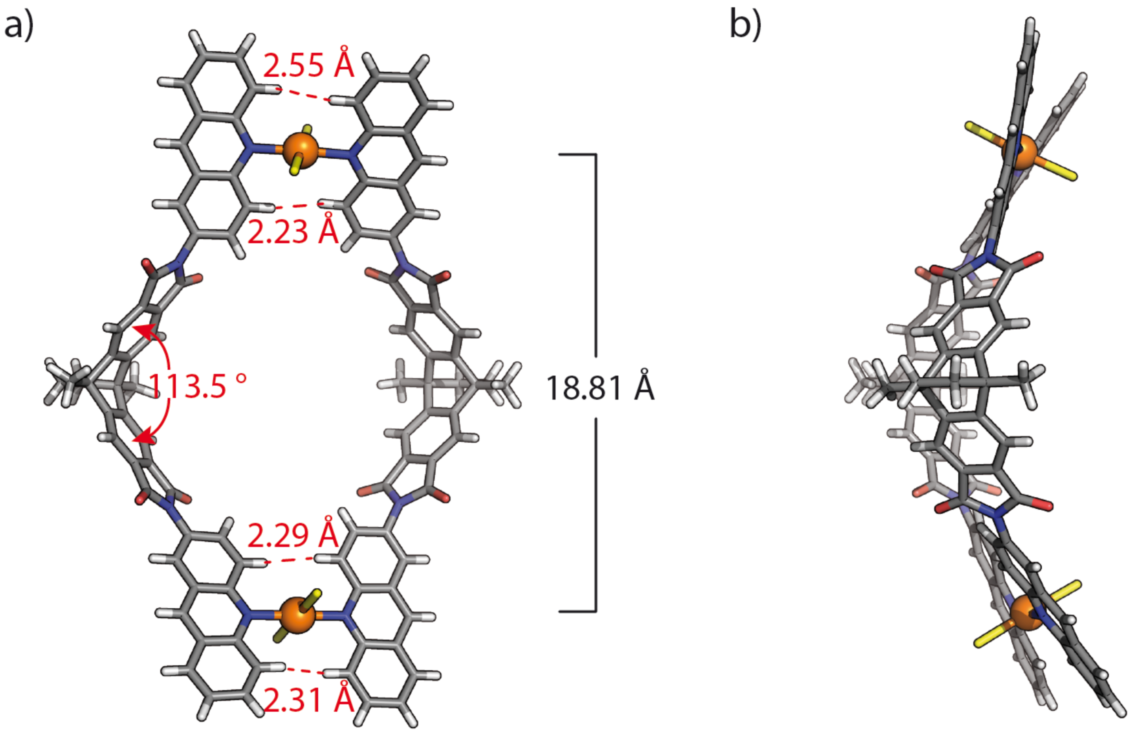

Figure S35 X-ray structure of [Pd_2**L**$^4{}_2Cl_4$]_B: (a) full structure showing the Pd–Pd distance of 18.81 Å; (b) side view. Color scheme: H, light grey; C, dark grey; N, blue; O, red; Cl, yellow; Pd, orange.

Table S10 Structural details involved in [Pd_2**L**$^4{}_2Cl_4$]_B.

Residues No.	Dihedral angle (°) between the backbone's benzene planes C16_C17_C18_C22_C23_C24 and C7_C8_C9_C13_C14_C15	Esd (°)	Dihedral angle (°) between planes N29_Pd1_Pd2 and N43_Pd1_Pd2	Esd (°)
2	66.221	0.411	29.047	0.451
3	66.774	0.287	28.327	0.423
Average	66.5		28.7	

5.6 Calculation of cavity volumes

Crystallographically determined structures of [C_{70}@Pd_2**L**$^2{}_4$]$^{4+}$ and [Pd_2**L**$^3{}_4$]$^{4+}$ were symmetry expanded and C_{70}, solvent molecules as well as BF_4^- counter ions were removed. Resulting inner cavities were calculated with VOIDOO[13] using a primary grid and plot grid spacing of 0.1 Å and 10 cycles of volume refinement with the size probe radius of 3.2 Å, the minimum radius such that it would not exit the cavity of this series of structures. Molecular visualization was done using PyMol.[14]

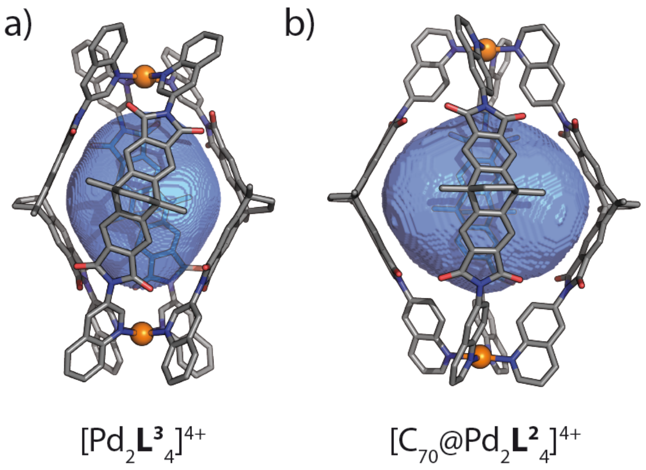

Figure S36 VOIDOO-calculated void space as shown (blue mesh) within the corresponding crystal structures for (a) cage $[Pd_2\mathbf{L^3}_4]^{4+}$ (518 Å^3) and (b) cage $[C_{70}@Pd_2\mathbf{L^2}_4]^{4+}$ (995 Å^3). Color scheme: C, dark grey; N, blue; O, red; Pd, orange.

6 Computational studies

All models shown below were constructed using Wavefunction SPARTAN '14[15] and first optimized on semiempiric PM6 level of theory without constraints. The resulting structures were then further refined by DFT structure optimization (B3LYP/C, H, N, O = 6-31g(d)/Pd LANL2DZ) using GAUSSIAN 09.[16]

6.1 DFT calculation of the energy change during the conversion from $[Pd_2L^2_3(MeCN)_2]^{4+}$ to $[Pd_2L^2_4]^{4+}$

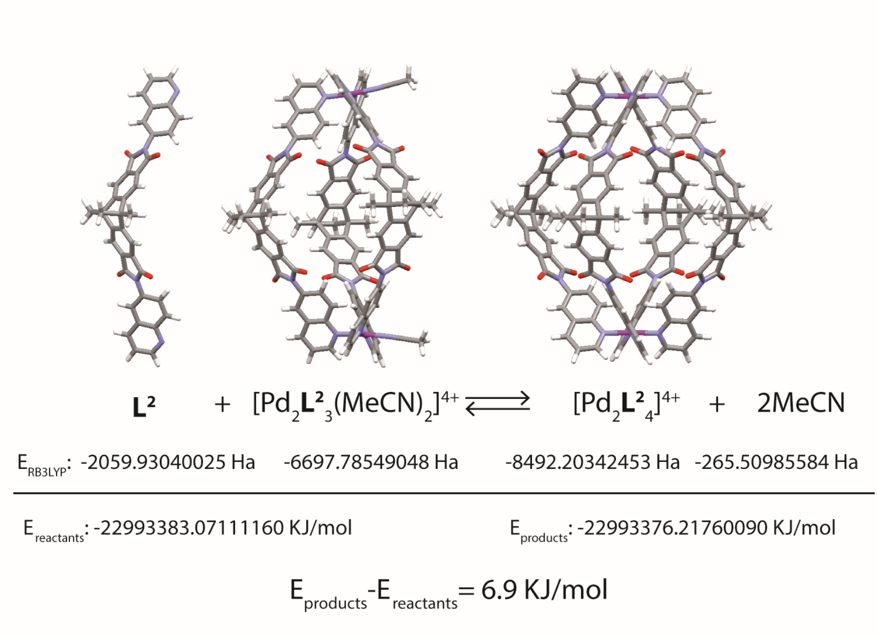

Figure S37 Scheme showing the optimized DFT structures of $\mathbf{L^2}$, $[Pd_2\mathbf{L^2}_3(MeCN)_2]^{4+}$ and $[Pd_2\mathbf{L^2}_4]^{4+}$. Calculated energies obtained from the geometry optimized structures are given below. The computed energy difference for the formation of $[Pd_2\mathbf{L^2}_4]^{4+}$ from $\mathbf{L^2}$ and $[Pd_2\mathbf{L^2}_3(MeCN)_2]^{4+}$ is positive and supports the experimental finding.

6.2 DFT calculation of the energy change during the conversion from $[Pd_2L^3_3(MeCN)_2]^{4+}$ to $[Pd_2L^3_4]^{4+}$

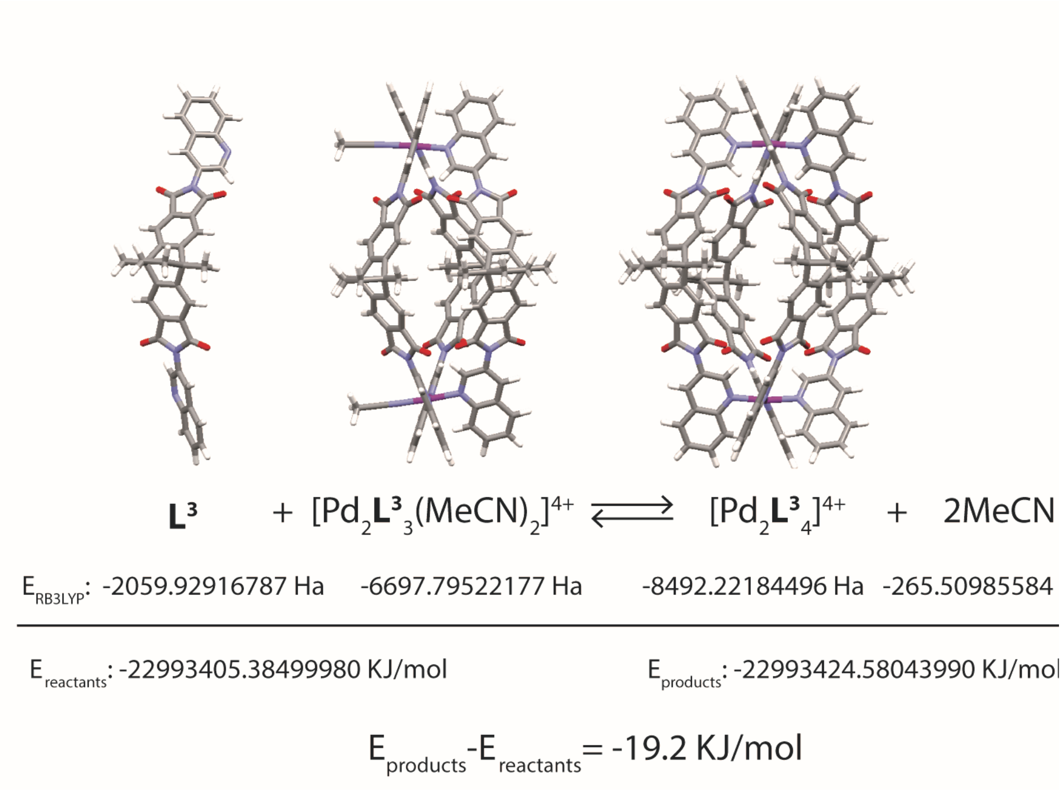

Figure S38 Scheme showing the optimized DFT structures of **L**^{3}, $[Pd_2$**L**$^3_3(MeCN)_2]^{4+}$ and $[Pd_2$**L**$^3_4]^{4+}$. Calculated energies obtained from the geometry optimized structures are given below. The energy difference for the formation of $[Pd_2$**L**$^3_4]^{4+}$ from **L**^{3} and $[Pd_2$**L**$^3_3(MeCN)_2]^{4+}$ is negative and supports the experimental finding.

6.3 Comparison of the DFT minimized energies of *cis*-$[Pd_2L^4{}_2(MeCN)_4]^{4+}$ and *trans*-$[Pd_2L^4{}_2(MeCN)_4]^{4+}$

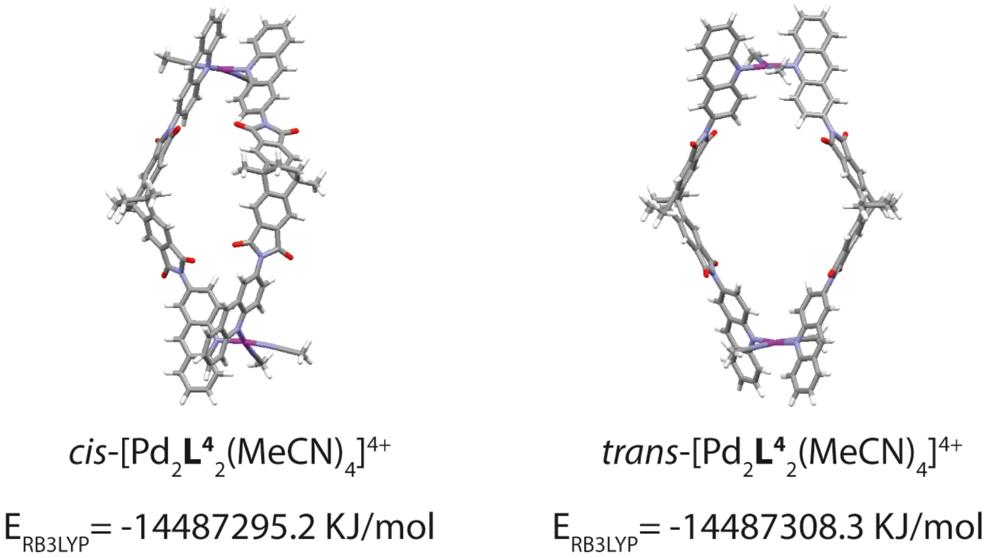

Figure S39 DFT energy minimized structures of tentative *cis*-$[Pd_2\mathbf{L^4}_2(MeCN)_4]^{4+}$ and observed *trans*-$[Pd_2\mathbf{L^4}_2(MeCN)_4]^{4+}$. According to the computed energies, *trans*-$[Pd_2\mathbf{L^4}_2(MeCN)_4]^{4+}$is 13.1 kJ/mol lower in energy.

7 References

[1] B. Chen, J. J. Holstein, S. Horiuchi, W. G. Hiller, G. H. Clever, *J. Am. Chem. Soc.* **2019**, *141*, 8907-8913.

[2] Y. Rogan, R. Malpass-Evans, M. Carta, M. Lee, J. C. Jansen, P. Bernardo, G. Clarizia, E. Tocci, K. Friess, M. Lanč, N. B. McKeown, *J. Mater. Chem. A* **2014**, *2*, 4874-4877.

[3] A. Burkhardt, T. Pakendorf, B. Reime, J. Meyer, P. Fischer, N. Stube, S. Panneerselvam, O. Lorbeer, K. Stachnik, M. Warmer, P. Rodig, D. Gories, A. Meents, *Eur. Phys. J. Plus* **2016**, *131*, 1-9.

[4] W. Kabsch, *Acta Crystallogr. Sect. D* **2010**, *66*, 125-132.

[5] G. Sheldrick, *Acta Crystallogr. Sect. A* **2015**, *71*, 3-8.

[6] G. Sheldrick, *Acta Crystallogr. Sect. C* **2015**, *71*, 3-8.

[7] C. B. Hubschle, G. M. Sheldrick, B. Dittrich, *J. Appl. Crystallogr.* **2011**, *44*, 1281-1284.

[8] D. Kratzert, J. J. Holstein, I. Krossing, *J. Appl. Crystallogr.* **2015**, *48*, 933-938.

[9] A. Thorn, B. Dittrich, G. M. Sheldrick, *Acta Crystallogr. Sect. A* **2012**, *68*, 448-451.

[10] D. Kratzert, I. Krossing, *J. Appl. Crystallogr.* **2018**, *51*, 928-934.

[11] A. Spek, *Acta Crystallogr. Sect. C* **2015**, *71*, 9-18.

[12] A. Spek, *Acta Crystallogr. Sect. D* **2009**, *65*, 148-155.

[13] G. J. Kleywegt, T. A. Jones, *Acta Crystallogr. Sect. D* **1994**, *50*, 178-185.

[14] W. L. DeLano, DeLano Scientific LLC, San Carlos (USA).

[15] Spartan ’08 Version 1.2.0, Wavefunction, Inc., Irvine (USA), **2009**.

[16] M. J. Frisch, et al., Gaussian09, Gaussian Inc., Wallingford (USA), **2009**.
